# Unlocking ginsenosides’ therapeutic power with polymer-based delivery systems: current applications and future perspectives

**DOI:** 10.3389/fphar.2025.1629803

**Published:** 2025-07-10

**Authors:** Xiaomin Yu, Yun Lu, Jiajing Chen, Yuqian Deng, Huan Liu

**Affiliations:** ^1^ Chengdu University of Traditional Chinese Medicine, Chengdu, China; ^2^ Hospital of Chengdu University of Traditional Chinese Medicine, Chengdu, China

**Keywords:** ginsenosides, polymer-based drug delivery system, bioavailability enhancement, artificial intelligence, drug delivery system design 1. introduction

## Abstract

Ginsenosides, as the main active ingredient of Panax plants, have been found to have extensive pharmacological activity and clinical therapeutic potential in recent years. However, its inherent physical and chemical properties such as poor solubility and low intestinal permeability result in low bioavailability, severely limiting its clinical application and translation. To address these challenges, polymeric carriers—valued for their excellent biocompatibility, structural tunability, and intelligent response functions—have been engineered to: (i) enhance solubilization *via* polymer conjugation and amphiphilic micellar encapsulation; (ii) achieve passive (EPR-mediated) and active (ligand-directed) tumor targeting to minimize off-target distribution; and (iii) enable on-demand drug release through pH-, ROS-, temperature-, and enzyme-responsive designs. In this review, we delve into the mechanistic principles and synergistic interactions underlying each functional module within a cohesive, function-centred design roadmap. Finally, we explore emerging interdisciplinary directions—including AI-guided polymer design, logic-gated nanocarriers, and microfluidic personalized fabrication—that promise to accelerate the bench-to-bedside translation of multifunctional ginsenoside therapeutics.

## 1 Introduction

Ginseng (Panax spp.; Araliaceae) has constituted a cornerstone of East-Asian medical practice for more than two millennia. The species *P. ginseng* C.A. Meyer, *P. quinquefolius* L., and *P. notoginseng* are the most extensively investigated representatives of the genus. Systematic phytochemical investigations initiated in the mid-twentieth century identified triterpenoid saponins—collectively termed ginsenosides—as the principal bioactive constituents (Kang and Min, 2012; Kim, 2012; Kiefer and Pantuso, 2003). By modulating intracellular signaling pathways, metabolic homeostasis, and immune function, ginsenosides furnish a mechanistic link between traditional empirical usage and contemporary biomedical science (Ratan et al., 2021; Kim et al., 2017; Lee and Kim, 2014; Kim et al., 2013).

Structurally, ginsenosides are classified into two major groups—protopanaxadiol (PPD) and protopanaxatriol (PPT). To date, more than 150 congeners have been isolated through solvent extraction, macroporous-resin adsorption, and preparative chromatographic techniques. These molecules demonstrate a diverse pharmacological repertoire, including anti-inflammatory, antioxidant, neuroprotective, and antineoplastic effects. Nonetheless, their clinical translation is impeded by significant biopharmaceutical limitations: oral bioavailability rarely surpasses 5%, owing to poor aqueous solubility, extensive gastrointestinal hydrolysis, and first-pass metabolism. Moreover, the intrinsic lipophilicity of several ginsenosides restricts their selective accumulation in target tissues, thereby constraining therapeutic efficacy (Jin et al., 2019).

Advanced drug-delivery systems (DDSs) have been developed to mitigate these challenges. Widely employed platforms encompass inorganic nanomaterials (e.g., mesoporous silica), liposomes, dendrimers, and a spectrum of natural and synthetic polymers. Inorganic carriers offer high drug-loading capacities but frequently exhibit suboptimal biodegradability, whereas liposomes improve solubility yet suffer from limited colloidal stability and shallow tissue penetration. Hybrid constructs—such as lipid–polymer nanoparticles—endeavour to integrate the complementary strengths of individual materials to enhance overall delivery performance.

Among available carriers, polymers are distinguished by their exceptional physicochemical tunability and functional versatility. Firstly, polymers can be sourced from naturally occurring macromolecules—such as chitosan, hyaluronic acid, gelatin, and alginate—which are extracted from renewable biomass and inherently offer excellent biocompatibility, enzymatic degradability, and specific biological recognition motifs that support mucosal adhesion and receptor-mediated uptake. Secondly, a wide array of synthetic polymers—including poly(lactic-co-glycolic acid) (PLGA), polyethylene glycol (PEG), polycaprolactone (PCL), and a growing library of stimulus-responsive block copolymers—can be manufactured through controlled polymerisation techniques, allowing precise regulation of molecular weight distribution, stereochemistry, and functional-group density. By judicious copolymerisation or post-synthetic modification, these natural and synthetic building blocks can be blended or grafted to form hybrid networks that combine the biosafety of natural polymers with the mechanical strength, programmable degradation kinetics, and environmental responsivity of their synthetic counterparts. The resulting polymer spectrum encompasses polysaccharides, proteins, polyesters, and smart architectures whose chain topology, cross-link density, and surface chemistry can be molecularly engineered to satisfy the pharmacokinetic and pharmacodynamic demands of oral, transdermal, injectable, intranasal, or receptor-targeted administration. Such bespoke design enables enhanced aqueous solubility, protection of labile ginsenosides from premature hydrolysis, on-demand release modulated by pH, redox, or enzymatic cues, and refined tissue specificity *via* ligand decoration or biomimetic camouflaging.

In this review, we systematically examine polymer-based strategies for unlocking the therapeutic potential of ginsenosides by organizing our discussion around three core functional objectives: **solubility enhancement**, **targeting**, and **stimuli-responsiveness**. First, we explore how polymer conjugation and amphiphilic block copolymers improve ginsenoside aqueous solubility and bioavailability. Next, we detail both passive and active tumor-targeting mechanisms—ranging from EPR-mediated accumulation to ligand-functionalized carriers—that enable preferential delivery to malignant tissues. Finally, we analyze a spectrum of stimuli-responsive systems, including pH-, ROS-, temperature-, and enzyme-triggered release platforms, highlighting how each design leverages unique tumor microenvironment cues. By structuring the manuscript in this way, we aim to provide a cohesive narrative that clarifies the mechanistic distinctions underlying each approach and suggests integrated pathways for future multifunctional carrier development.

## 2 Categories and application of ginsenosides

**FIGURE 1:**
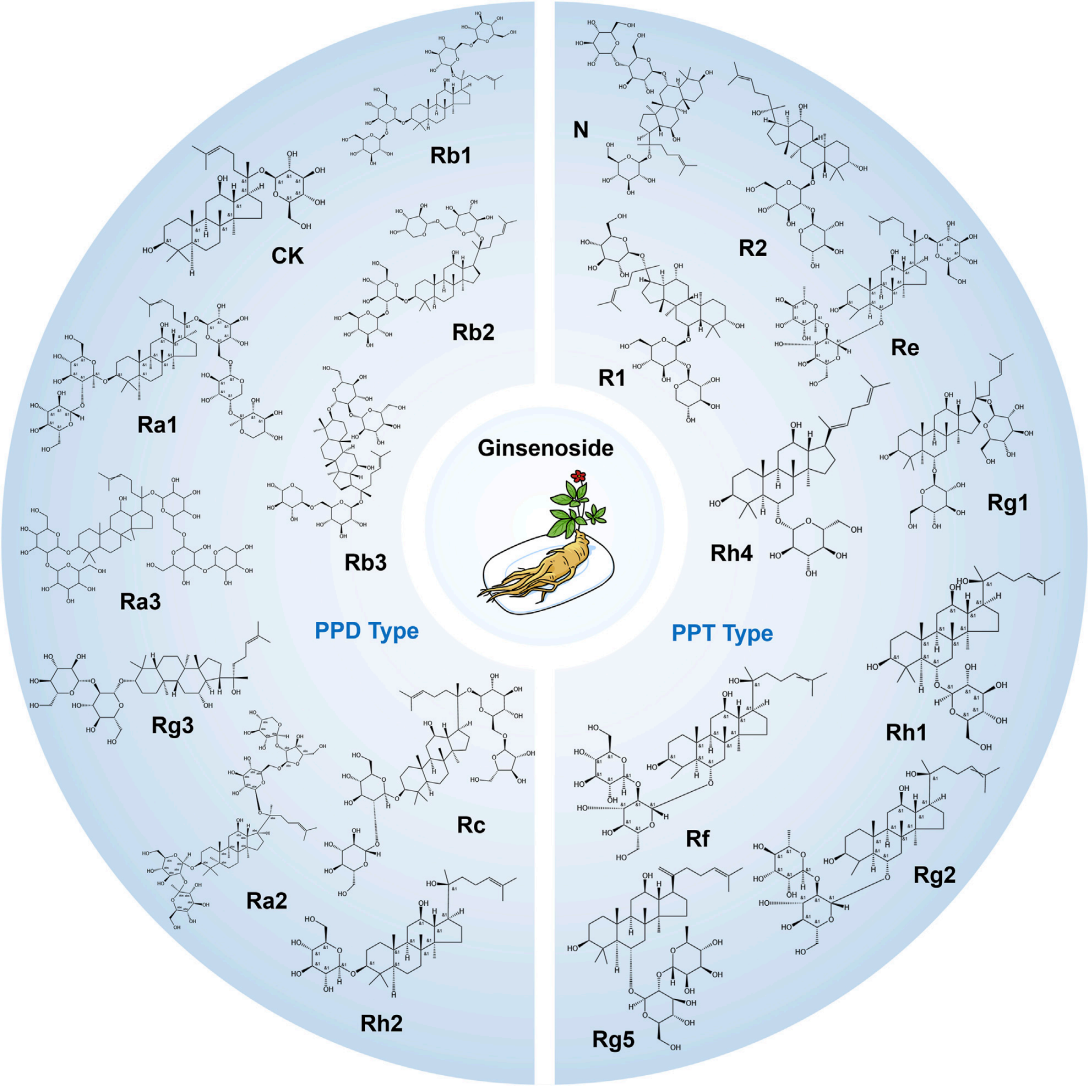
The molecular structures of representative ginsenosides of PPD and PPT types.

**FIGURE 2:**
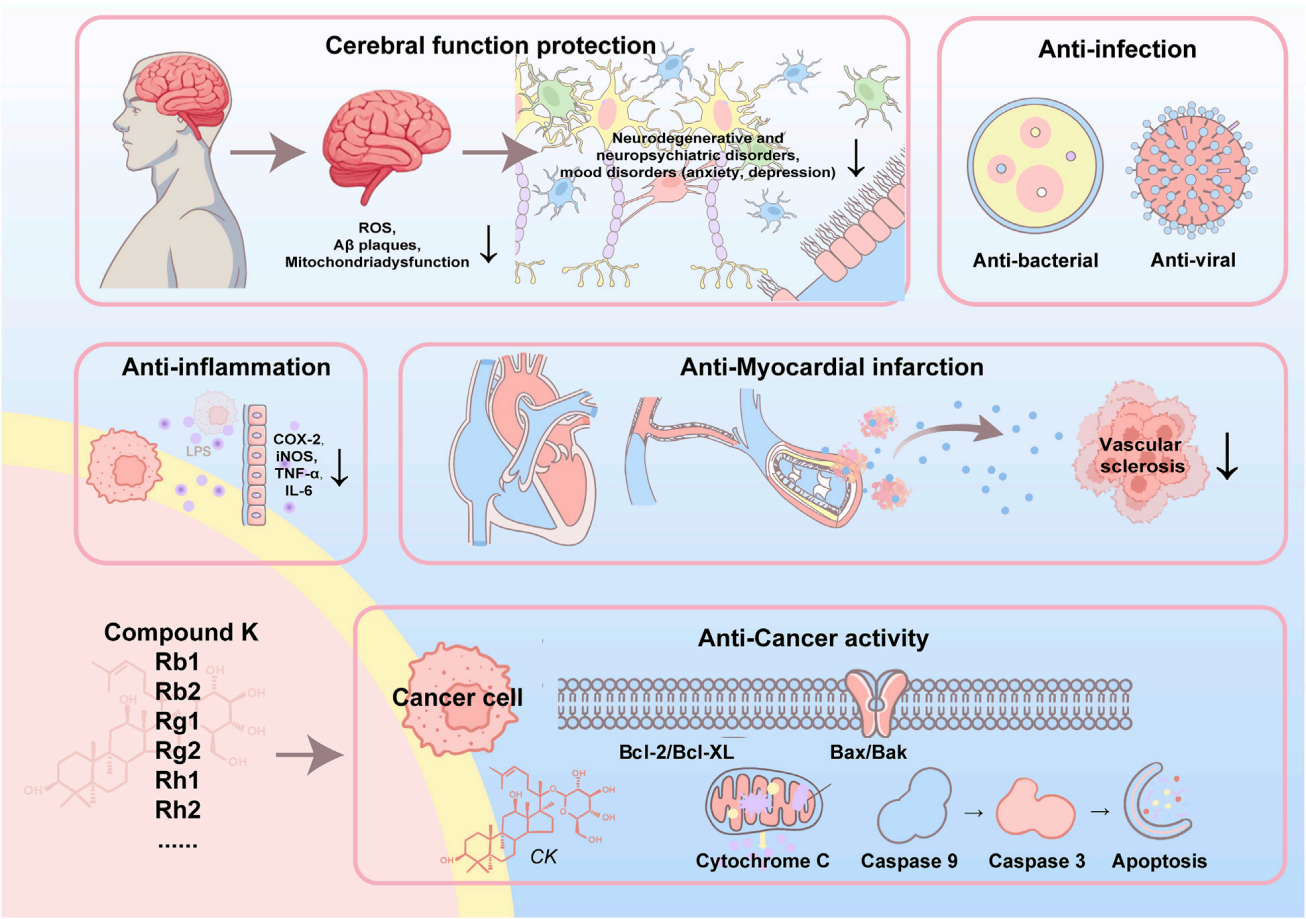
Representative therapeutic applications of various ginsenosides.

Ginsenosides are a class of steroidal saponins derived from the Panax ginseng plant (Fuzzati, 2004; Christensen, 2009). Most ginsenosides are considered pharmacologically benign or even beneficial as general tonics, exhibiting low toxicity (Zuo et al., 2022). They are classically grouped into four structural families (Christensen, 2009; Hu et al., 2024). Protopanaxadiol (PPD) ginsenosides—typified by Rb1, Rb2, Rc and Rd—share a dammarane tetracyclic aglycone bearing glycosyl moieties mainly at the C-3 and C-20 hydroxyls. Protopanaxatriol (PPT) ginsenosides, represented by Rg1, Re and Rg2, contain an additional C-6 hydroxyl, so sugars are usually anchored at C-6 and C-20 (Li et al., 2022). Less abundant oleanane-type saponins (e.g., Ro) feature an oleanolic-acid backbone, whereas ocotillol-type ginsenosides (e.g., F11) harbor an epoxy ring at C-20(21). Standard aqueous or hydro-ethanolic extraction followed by macroporous-resin enrichment and preparative HPLC can efficiently partition PPD- and PPT-rich fractions; controlled acid/thermal treatment of PPD congeners furnishes pharmacologically prized derivatives such as Rg3, and site-selective glycosyltransferases enable analogous transformations within the PPT series. Owing to their heavy glycosylation and rigid triterpenoid core, all ginsenosides dissolve poorly in water, contributing to their notoriously low oral bioavailability. Pharmacologically, ginsenosides demonstrate a broad spectrum of activities. PPD-type ginsenosides, such as Rb_1_ and Rd, have been implicated in anti-inflammatory, immunomodulatory, and neuroprotective effects (Liu et al., 2019; Son et al., 2016; Song et al., 2020; Wu et al., 2021; Qian et al., 2019). PPT-type ginsenosides, like Rg1 and Re, are known for their cognitive-enhancing, anti-fatigue, and cardioprotective properties (Gao et al., 2020; Qu et al., 2011; Wang et al., 2014; Kenarova et al., 1990). Furthermore, ginsenosides can influence various other signaling cascades, thereby exerting diverse biological effects. Figure 1 shows the molecular structures of 20 types of ginsenosides. **2.1–2.9** elaborated on the pharmacological activities and therapeutic applications of nine representative ginsenosides. Figure 2 summarize the representative applications of various ginsenosides, Table 1 summarizes the effects and mechanisms of more ginsenosides.

**TABLE 1:** Summary of the molecular mechanisms of action of the main components of ginsenosides and their potential application scenarios.

Categories	Ginsenoside	Pharmacological mechanism of action	Potential therapeutic applications	References
PPD type	*Compound K*	Modulates metabolic pathways; exerts significant anti-inflammatory and antioxidant effects; lowers levels of proinflammatory cytokines; improves insulin sensitivity, and displays anti-apoptotic activity; anti-aging and neuroprotective properties.	Metabolic disorders; inflammatory conditions; osteoporosis; neurodegenerative diseases; aging.	Thangavelu et al. (2020), Liu et al. (2022a), Yan et al. (2014), Yang et al. (2015)
	*Rb1*	neuroprotection; anti-inflammatory and anti-apoptotic; glucose and lipid metabolism regulation; insulin sensitivity improvement.	Central nervous system injuries; neurodegenerative diseases; type 2 diabetes and metabolic syndrome; cardiovascular and cerebrovascular recovery.	Ling et al. (2024), Gong et al. (2022), Shi et al. (2020), Jiang et al. (2025), Zhuang et al. (2025), Zhou et al. (2019), Zou et al. (2022)
	*Rb2*	insulin sensitivity improvement; glucose metabolism modutalion; antioxidant and anti-inflammatory effects, osteoblast function promotion; antitumor activity; anti-photoaging activity; potential antiviral actions.	Metabolic dysregulation ischemic injury; osteoporosis prevention; adjunctive tumor therapy; prevention of photoaging; enhancement of immune and antiviral defenses.	Dai et al. (2018), Choi et al. (2013), Liu et al. (2020), Ma et al. (2024), Li et al. (2024), Sato et al. (1994), Dai et al. (2019), Oh et al. (2015), Yoo et al. (2013), Nguyen and Nguyen (2019)
	*Rg3*	anti-inflammatory and antioxidant properties; neuronal apoptosis inhibition; neuroinflammation modulation; immune responses modulation; regulation of pathways related to mood; antifibrotic activity.	Various cancers; neurodegenerative diseases and brain injuries; cardiovascular diseases; metabolic syndrome; viral hepatitis.	Wang et al. (2015), Kim et al. (2009), Xie et al. (2017), K et al., 2013; Li et al. (2021), Hwang et al. (2009), Rhim et al. (2002), Lee et al. (2013), Oh et al. (2022), Tang et al. (2018), Kim et al., 2012a; Nah (2014), Chang et al. (2023), Cheng and Li (2016), Lee et al. (2020), Wang et al. (2024)
	*Ra1*	Protein tyrosine kinase (PTK) inhibition; Signaling pathway modulation (MAPK/ERK, PI3K/Akt/mTOR, and NF-κB)	Coronary Artery Disease (CAD); Immune Regulation; Anti-cancer.	Ma et al. (2020)
	*Rb3*	regulates energy metabolism and apoptosis;	antidepressant-like effects; ischemia-reperfusion injury reduction;	Cui et al. (2012), Chen et al. (2019), Liu et al. (2014)
	*Rc*	SIRT1 Activator; anti-inflammatory activity; SIRT6, PPARα/γ, PGC-1α, and AMPK activation	improve cardio- and neuroprotective functions; anti-inflammatory actions; metabolic syndrome.	Huang et al. (2021), Yu et al. (2017), Lu et al. (2023)
	*Rh2*	inducing apoptosis and cell-cycle arrest in cancer cells; anti-inflammatory activities; multiple signaling pathways and ion channels regulation.	Various malignancies; chronic inflammatory and autoimmune diseases; central nervous system disorders.	Liu et al. (2022b), Hu et al. (2023), Li et al. (2020a), Chung et al. (2013), Oh et al. (1999), Li et al. (2023), Ben-Eltriki et al. (2023), Li et al. (2015), Xiaodan and Ying (2022), Shi et al. (2022), Hou et al. (2018)
PPT type	*Rg1*	promoting cancer cell apoptosis cell-cycle arrest; anti-inflammatory and antioxidant properties, neuroinflammation and apoptosis reduction; enhancing insulin sensitivity.	Neurological diseases; inflammation-related conditions; type 2 diabetes; hepatic fibrosis.	Kim et al. (2011), Fan et al. (2024), Xie et al. (2023), Zhang et al. (2016), Yang et al. (2022), Jiang et al. (2020), Wang et al. (2021a), Fan et al. (2019), Liu et al. (2017), Li et al. (2014), Wei et al. (2018), Lin et al. (2020), Chen et al. (2003), Lee et al. (2016)
	*Rg2*	anti-inflammatory and immunomodulatory properties; antioxidant activity; reducing neuronal oxidative reduction; dopamine signaling modulation.	Neurodegenerative and neuropsychiatric disorders; mood disorders (anxiety, depression).	Sugimoto et al. (2009), Zhang et al. (2025), Fu et al. (2018), Ren et al. (2017), Gao et al. (2019)
	*Rh1*	inducing apoptosis and cell-cycle arrest; immune responses modulation; mitigating central inflammation and oxidative stress.	Inflammatory diseases; neurodegenerative disorders; cancer; cardiovascular diseases.	Liu et al. (2023a), Piao et al. (2020), Tam et al. (2018), Park et al. (2004), Huynh et al. (2021), Lee et al. (2003), Xu et al. (2022)
	*Rg5*	antioxidant, anti-inflammatory, and anti-apoptotic activities; neuronal oxidative stress reduction.	Malignant tumors; inflammatory diseases; neurological disorders; diabetes and its complications.	Cheng et al. (2023), Elsaman et al., 2024; Lee (2014), Kim et al. (2012b), Chu et al. (2014), Gao et al. (2022a), Zhu et al. (2020)
	*R2*	NF-κB inhibition	Anti-inflammatory effects	Jeong et al. (2015)
	*Re*	NF-κB inhibition; PPARγ activation; suppression of hepatic gluconeogenesis; MAPK activation	Anti-Diabetes; Anti-Nerve Injuries; Anti-Inflammatory Effects; Anti-Cardiovascular Diseases	Gao et al. (2022b), Peng et al. (2012), Xie et al. (2005)
	*R1*	lipid regulation;	Improve cardiovascular health; Anti-inflammatory and antioxidant;	Zhang et al. (2020a)

### 2.1 Compound K

Ginsenoside compound K (CK), also referred to as 20-O-β-D-glucopyranosyl-20(S)-protopanaxadiol, is a tetracyclic triterpenoid with the molecular formula C_36_H_62_O_8_ and a molecular weight of approximately 622–623 g/mol. It is a rare protopanaxadiol-type saponin generated *via* the hydrolytic cleavage of major ginsenosides such as Rb1, Rb2, and Rc by human gut microbiota or other biochemical transformations (Thangavelu et al., 2020; Liu T. et al., 2022; Yan et al., 2014). In contrast to primary ginsenosides, CK exhibits superior bioavailability owing to its smaller molecular weight and fewer sugar moieties, which enhance its intestinal absorption and systemic circulation (Yang et al., 2015).

Recent investigations have demonstrated CK’s broad pharmacological benefits against metabolic diseases, including obesity, hyperlipidemia, type 2 diabetes, and non-alcoholic fatty liver disease. Mechanistically, CK modulates various signaling pathways—notably AMPK, PPARγ, SIRT1, Wnt, NF-κB, and TP53—thereby influencing energy metabolism, adipogenesis, inflammation, and insulin sensitivity. Through its capacity to reduce inflammatory cytokines (such as TNF-α and IL-6) and reactive oxygen species, CK alleviates tissue injury and fosters an anti-inflammatory microenvironment. In preclinical models, CK has also shown protective effects in osteoporosis by enhancing osteoblast function, as well as neuroprotective and anti-aging properties *via* antioxidant and anti-apoptotic mechanisms.

Pharmacokinetic data indicate that CK’s plasma concentration peaks relatively late compared to other ginsenosides, underscoring the essential role of gut bioconversion in its availability. Limited clinical studies suggest that CK is well-tolerated in humans, with adverse events generally mild and self-limiting. Overall, CK’s potent bioactivities, broad therapeutic profile, and favorable safety characteristics make it a compelling candidate for managing metabolic disorders and beyond, warranting more extensive clinical research into its applications.

### 2.2 Rb1

Ginsenoside Rb1 is a representative protopanaxadiol (PPD) saponin derived mainly from Panax ginseng or Panax notoginseng. It generally appears as a white amorphous powder with the molecular formula approximately C_54_H_92_O_23_ and a molecular weight near 1,100 g/mol. Rb1 has relatively low water solubility but can be absorbed and metabolized into active intermediates (notably ginsenoside Rd and F2) in the gastrointestinal tract, which then exhibit multiple pharmacological effects.

Preclinical research shows that Rb1 exerts strong neuroprotective, antioxidant, anti-inflammatory, and anti-apoptotic properties (Ling et al., 2024; Gong et al., 2022). These effects underlie its demonstrated benefits in models of ischemic stroke (Shi et al., 2020), traumatic brain injury (Xie et al., 2021), and various neurodegenerative disorders (Jiang et al., 2025; Zhuang et al., 2025). Beyond the nervous system, Rb1 also helps regulate glucose metabolism and insulin sensitivity, suggesting potential anti-diabetic and cardiometabolic applications (Zhou et al., 2019; Zou et al., 2022). Although extensive clinical studies are still limited, existing data indicate that Rb1-based interventions may improve functional recovery in stroke patients, attenuate cognitive decline, enhance mood, and mitigate complications of metabolic syndrome. Such findings support broader investigation of ginsenoside Rb1 as a promising therapeutic agent.

### 2.3 Rb2

Ginsenoside Rb2 is a protopanaxadiol-type saponin found abundantly in ginseng stems and leaves. It is a relatively large molecule with low water solubility and limited oral bioavailability (Miao et al., 2022). Despite these absorption challenges, Rb2 demonstrates a broad therapeutic profile. In metabolic disorders, it helps regulate insulin sensitivity, glucose metabolism, and lipid accumulation—thereby improving conditions such as type 2 diabetes and obesity (Dai et al., 2018). It also exerts anti-inflammatory and antioxidant effects that benefit cardiovascular health (Choi et al., 2013), for example, by mitigating myocardial ischemia/reperfusion injury (Liu et al., 2020).

Rb2 supports bone integrity by reducing osteoclast-mediated resorption and promoting osteoblast function, which can help prevent osteoporosis (Ma et al., 2024; Li et al., 2024). Additionally, various *in vitro* and *in vivo* studies have shown that Rb2 can inhibit tumor growth and metastasis (Sato et al., 1994; Dai et al., 2019), protect against UV-induced skin photoaging (Oh et al., 2015), and possibly assist in treating viral infections (Yoo et al., 2013; Nguyen and Nguyen, 2019). Although more thorough clinical trials are needed, Rb2’s diverse pharmacological actions suggest significant promise for developing new interventions to manage diabetes and obesity, enhance cardiac function, strengthen bone health, combat certain cancers, and improve skin and immune system resilience.

### 2.4 Rg1

Ginsenoside Rg1 is a dammarane-type (protopanaxatriol) triterpenoid saponin isolated from Panax ginseng. It has a four-ring steroid-like core bearing sugar moieties that enhance its water solubility. Rg1 typically presents as a white to off-white amorphous powder with a molecular weight of around 800–801 g/mol (C_42_H_72_O_14_) (Kim et al., 2011). It is relatively stable under neutral conditions but can undergo hydrolysis in strongly acidic or alkaline environments (Fan et al., 2024).

This distinctive structure underpins Rg1’s noteworthy pharmacological properties. It has been extensively studied for anti-inflammatory and antioxidant effects that help combat oxidative stress in conditions such as metabolic disorders (Xie et al., 2023) and neurodegenerative diseases (Zhang et al., 2016; Yang et al., 2022). Preclinical research demonstrates Rg1’s neuroprotective capacity, showing promise in mitigating depression-like behavior and cognitive deficits by modulating pathways involved in neuroinflammation and oxidative injury (Jiang et al., 2020; Wang et al., 2021a). Additionally, Rg1 contributes to regulating glucose metabolism and insulin sensitivity, suggesting benefits in type 2 diabetes management by reducing hyperglycemia-induced inflammation and improving pancreatic β-cell function (Fan et al., 2019; Liu et al., 2017). Its hepatoprotective influence also reduces liver injury markers and fibrosis-related parameters (Li et al., 2014; Wei et al., 2018).

Beyond these organ-specific effects, Rg1 has been shown to inhibit apoptosis in multiple disease models while enhancing cell-survival signaling (Lin et al., 2020; Chen et al., 2003). It also exerts immunomodulatory properties that can bolster the body’s defense against pathogenic (Qu et al., 2011) or autoimmune insults (Lee et al., 2016). Altogether, Rg1’s amphiphilic nature, relative stability, and low toxicity profile make it a compelling candidate for further clinical development. As research progresses, Rg1 may emerge as a versatile adjunct or standalone therapy for managing diverse inflammatory, metabolic, and neurodegenerative conditions.

### 2.5 Rg2

Ginsenoside Rg2 is classified as a protopanaxatriol-type triterpenoid saponin found in Panax ginseng. It typically appears as an off-white powder with an approximate molecular formula C_42_H_72_O_13_ (Sugimoto et al., 2009). Rg2 exhibits moderate water solubility but dissolves more readily in ethanol-based solvents. Although present in relatively small quantities in natural ginseng, Rg2 has demonstrated noteworthy pharmacological activities in both cell-based and animal models.

Studies indicate that Rg2 exerts antioxidant, anti-inflammatory, and anti-apoptotic effects, contributing to neuroprotection in conditions such as Alzheimer’s disease (Zhang et al., 2025), vascular dementia (Liu S. et al., 2023), and cerebral ischemia–reperfusion injury (Fu et al., 2018). In these contexts, Rg2 helps stabilize synaptic structures and reduce oxidative stress (Liu S. et al., 2023). Rg2 has also shown promise in modulating neuroinflammation, alleviating anxiety- and depression-like behaviors, and improving cognitive deficits (Ren et al., 2017; Gao et al., 2019). While large-scale clinical trials are lacking, these preclinical findings suggest that Rg2 holds therapeutic potential for neurodegenerative and neuropsychiatric disorders, warranting further investigation into its clinical applications as a protective agent in age-related neurological conditions.

### 2.6 Rg3

Ginsenoside Rg3, a rare saponin predominantly found or generated from Panax ginseng (especially in steamed red ginseng), has attracted considerable attention for its multidimensional therapeutic effects. Existing in two stereoisomeric forms—20(S)-Rg3 and 20(R)-Rg3—this dammarane triterpenoid is primarily produced *via* heat/acid or enzymatic conversion of major PPD-type ginsenosides (e.g., Rb1, Rd) during ginseng processing or fermentation (Wang et al., 2015). Despite its naturally low abundance, Rg3 exhibits wide-ranging bioactivities by modulating key molecular pathways (including NF-κB (Kim et al., 2009), PI3K/Akt (Xie et al., 2017), JNK (K et al., 2013), MAPKs (Li et al., 2021), and AMPK (Hwang et al., 2009)) and interacting with multiple ion channels and receptors (Rhim et al., 2002; Lee et al., 2013).

Rg3’s anticancer properties are well documented: it can inhibit tumor growth, invasion, angiogenesis, and metastasis, often by promoting apoptosis, inducing cell-cycle arrest, and enhancing the efficacy of conventional chemotherapeutic agents (Oh et al., 2022; Tang et al., 2018; Kim J. W. et al., 2012). In parallel, Rg3 exerts protective actions against oxidative damage and inflammation in various contexts such as neurodegenerative disorders (Nah, 2014), acute organ injuries (Chang et al., 2023; Cheng and Li, 2016), and metabolic abnormalities (Lee et al., 2020). Notably, it can stabilize voltage-gated Ca^2+^ and Na^+^ channels and reduce excitotoxicity in neuronal models, guard against ischemic or arrhythmic cardiac events, and improve insulin sensitivity and lipid profiles in metabolic syndrome (Lee et al., 2020). In liver-related studies, Rg3 has shown hepatoprotective roles in conditions like viral hepatitis, alcoholic/non-alcoholic fatty liver disease, and fibrotic progression by suppressing inflammatory mediators, attenuating hepatic stellate cell activation, and bolstering antioxidant defenses (Wang et al., 2024). Although challenges remain—particularly in achieving high-yield production and optimizing delivery—Rg3’s low toxicity, proven efficacy, and potential synergy in combination therapies affirm its promise as a versatile candidate for further development in preventing or managing diverse human diseases.

### 2.7 Rg5

Ginsenoside Rg5 is a minor protopanaxadiol-type saponin formed during the high-temperature steaming of ginseng roots. It arises *via* the deglycosylation and subsequent dehydration of major ginsenosides such as Rb1 and Rg3. Rg5 generally appears as a white crystalline powder with a melting point around 188°C–190°C and features a dammarane skeleton bearing fewer sugar moieties than its precursors. Although not as abundant as major ginsenosides (e.g., Rg1 or Rb1), Rg5 exhibits comparatively higher lipophilicity, which facilitates its cellular uptake and has been linked to versatile biological actions (Cheng et al., 2023).

Indeed, considerable *in vitro* and *in vivo* evidence highlights the therapeutic promise of Rg5 across multiple disease domains. A leading area of interest is its anticancer potential: Rg5 promotes apoptosis, induces cell-cycle arrest, and inhibits proliferation in a range of tumor models, including breast, gastric, and colorectal cancers (Elsaman et al., 2024). It also displays anti-inflammatory and antioxidative properties, mitigating conditions such as renal and hepatic injury by dampening pro-inflammatory cytokine release and scavenging reactive oxygen species (Lee, 2014; Kim T. W. et al., 2012). Rg5 confers neuroprotective benefits as well, as seen in experimental models of neurodegeneration and ischemic injury—in part by regulating inflammatory pathways (e.g., NF-κB) and modulating apoptotic cascades (Chu et al., 2014; Gao X. F. et al., 2022). Further research has demonstrated Rg5’s capacity to improve insulin sensitivity and attenuate hyperglycemia-associated damage in diabetic settings (Zhu et al., 2020). Despite its potential, Rg5’s relatively low natural abundance has spurred the development of innovative extraction and formulation techniques—such as nanoparticle-based delivery—to enhance yields, stability, and bioavailability for eventual clinical translation.

### 2.8 Rh1

Ginsenoside Rh1 is a minor protopanaxatriol-type saponin derived from Panax ginseng, especially abundant in red ginseng after high-temperature processing. Structurally, Rh1 features the tetracyclic dammarane skeleton common to ginsenosides but with fewer attached sugar moieties than its major counterparts, and it often appears as a white crystalline powder (Piao et al., 2020). Rh1 is relatively more lipophilic compared to some other ginsenosides, which facilitates its interaction with cell membranes and may enhance its cellular uptake (Tam et al., 2018). However, studies have found that it has limited oral bioavailability—due in part to metabolism by gut flora and rapid systemic clearance—prompting interest in improving its delivery *via* nanoparticle or self-emulsifying systems.

Therapeutically, Rh1 demonstrates notable anti-inflammatory and immunomodulatory effects. It downregulates pro-inflammatory cytokines (e.g., TNF-α, IL-6) and inhibits key pathways like NF-κB and MAPKs in various models, offering potential benefits in conditions such as dermatitis, colitis, and autoimmune disorders (Park et al., 2004). Additionally, Rh1’s antioxidant properties stem from its ability to suppress excessive reactive oxygen species generation (Huynh et al., 2021). In neuronal cell lines, Rh1 exerts neuroprotective effects by mitigating oxidative stress and modulating dopaminergic signaling (Liu S. et al., 2023). In some cancer models (e.g., leukemia and glioma), Rh1 can induce differentiation of cancer cells, reduce expression of metastasis-related factors like MMP-9, and augment immune-mediated cytotoxicity, making it an intriguing adjunct in oncology (Tam et al., 2018). Rh1 also shows mild estrogenic activity and can affect platelet aggregation and cardiovascular processes such as endothelial adhesion (Lee et al., 2003; Xu et al., 2022). Overall, Rh1’s favorable safety profile, broad pharmacological range, and amenability to enhanced delivery formulations highlight its promise as a multitarget agent for inflammatory, immune, neurodegenerative, and neoplastic diseases.

### 2.9 Rh2

Ginsenoside Rh2 is a notable protopanaxadiol-type saponin that typically arises in processed ginseng (such as red ginseng) or through the enzymatic conversion of larger ginsenosides like Rg3 (Liu L. et al., 2022). Structurally, Rh2 has a dammarane steroid backbone with only one or two sugar units, resulting in poor water solubility but comparatively greater membrane permeability than highly glycosylated ginsenosides (Liu L. et al., 2022). Rh2 has garnered attention for its potent anticancer and immunomodulatory activities. Multiple studies report that Rh2 induces apoptosis and cell-cycle arrest in cancer cells, while inhibiting tumor invasion, angiogenesis, and metastasis (Hu et al., 2023; Li X. et al., 2020). It has shown efficacy against a variety of cancers—including leukemia (Chung et al., 2013) and cancers of the breast (Oh et al., 1999), colon (Li et al., 2023), and prostate (Ben-Eltriki et al., 2023)—often enhancing the effectiveness of chemotherapy drugs and even helping to reduce their toxicity by sensitizing tumor cells to treatment. Beyond its direct antiproliferative effects, Rh2 exerts anti-inflammatory actions: it can suppress excessive inflammatory responses (for example, by downregulating the NF-κB pathway and pro-inflammatory cytokines) and has demonstrated benefits in models of chronic inflammation and autoimmune reactions (Li et al., 2015). Interestingly, Rh2 also appears to modulate the immune system; it can boost certain immune surveillance mechanisms that help the body target cancer cells, while simultaneously protecting normal tissues from inflammation-induced damage (Xiaodan and Ying, 2022). In neurological contexts, preliminary research suggests Rh2 may offer neuroprotective and antidepressant-like effects, possibly by reducing neuroinflammation and oxidative stress in the brain (Shi et al., 2022; Hou et al., 2018). Like other PPD-type ginsenosides, Rh2 is generally well tolerated in preclinical studies, with low toxicity. Its clinical application, however, is limited by low natural abundance and suboptimal pharmacokinetics. As a result, ongoing efforts aim to improve Rh2’s delivery and bioavailability—through methods like chemical structure modification and advanced nanoparticle formulations—to fully harness its therapeutic potential.

## 3 Polymer-based drug systems

Despite their robust pharmacological profiles, many ginsenosides exhibit poor water solubility, which drastically reduces their bioavailability. They also undergo rapid metabolism and elimination, leading to low systemic exposure. In addition, limited membrane permeability and susceptibility to enzymatic degradation hinder ginsenosides from accumulating in target tissues, often necessitating higher or more frequent dosing.

To address these issues, various drug delivery approaches—such as nanoparticle encapsulation, micellar formulations, and polymer–drug conjugates—have been explored for ginsenosides (Dai et al., 2016; Hong et al., 2019). These strategies aim to improve solubility and stability, prolong circulation time, and enable controlled or targeted release of the active compound. Increasingly sophisticated designs, including pH-sensitive bonds, thermogelling polymers, and ligand-based targeting, further enhance localization of ginsenosides to the desired site of action while minimizing off-target effects. Polymeric carriers in particular offer unparalleled versatility: researchers can fine-tune their chemical composition, molecular weight, and functional groups to precisely control drug loading capacity, release kinetics, and targeting capabilities (Sung and Kim, 2020; Al-Tahami and Singh, 2007). Many polymers commonly used for drug delivery (e.g., PLGA, chitosan, PEG) are biocompatible and biodegradable, which facilitates their regulatory approval for medical use.

By integrating stimuli-responsiveness (triggering release in response to pH, temperature, or redox conditions), attaching targeting ligands, or even incorporating cell membrane coatings for biomimicry, polymeric systems can overcome multiple biological barriers simultaneously. Moreover, these carriers are often amenable to scalable manufacturing and can co-deliver multiple therapeutic agents, paving the way for combination therapies. Polymeric nanocarriers also tend to exhibit improved pharmacokinetics and preferential accumulation in disease sites *via* the enhanced permeability and retention (EPR) effect. Collectively, these features make polymer-based drug delivery a particularly promising avenue for maximizing the therapeutic potential of ginsenosides.

## 4 Polymer-based systems for protection and solubility enhancement

Basic polymer-based delivery systems typically focus on a single primary enhancement—often improving the solubility and stability of ginsenosides in aqueous media, or modulating their release rate. Such formulations employ biocompatible polymers (natural or synthetic) to encapsulate or solubilize ginsenosides, yielding nanoscale carriers (e.g., nanoparticles, micelles) or depot matrices (e.g., microparticles, films, hydrogels) that address core issues like poor dissolution and rapid clearance of the drug (Sharma and Garg, 2010; Dragojevic et al., 2015).

### 4.1 Natural polymer-based DDS

**FIGURE 3:**
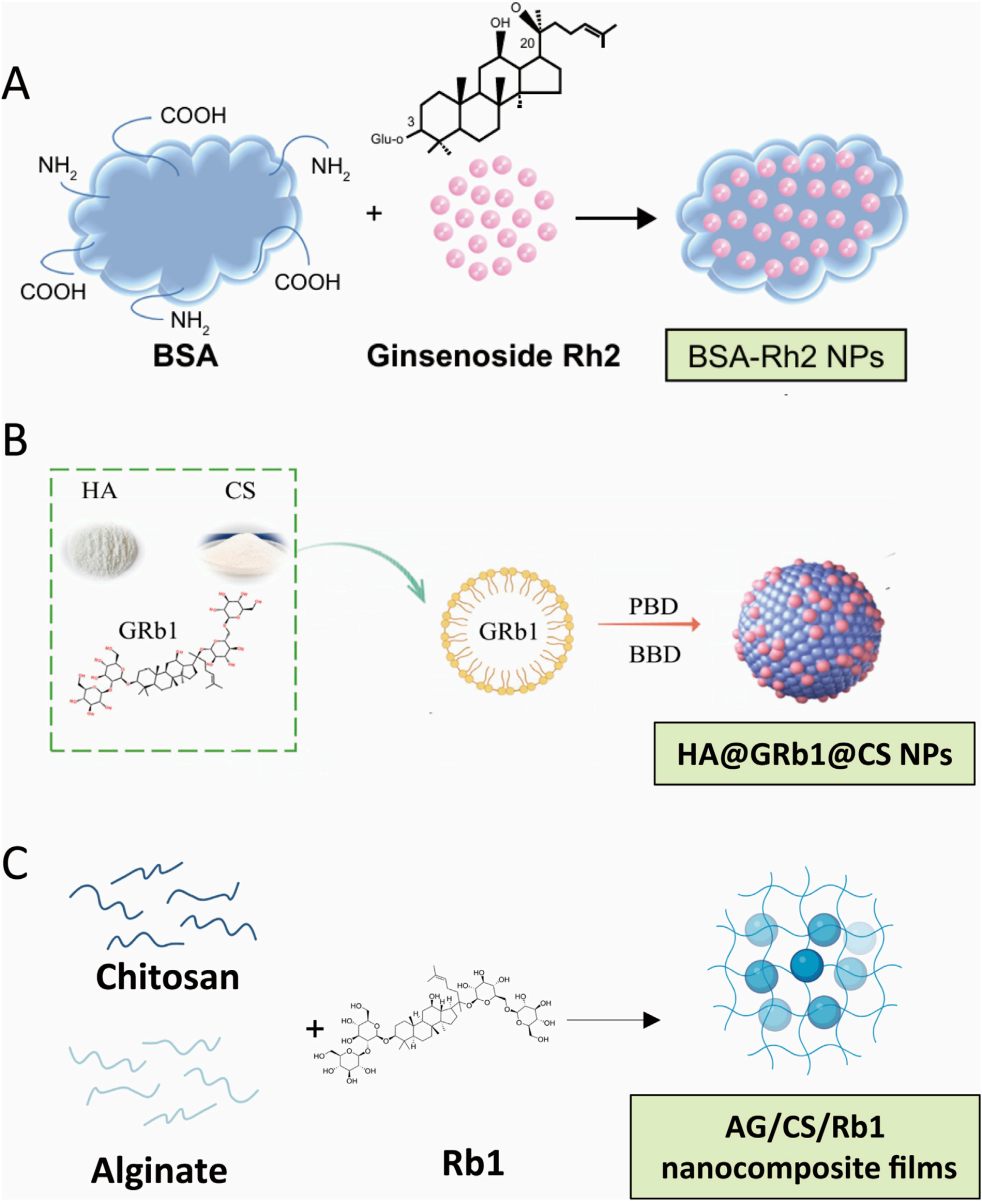
Representative Natural polymers based-ginsenoside delivery system. **(A)** Scheme for the preparation of BSA-Rh2 NPs. Reproduced with permission from Singh et al. (2017). Copyright ^©^ Dove Medical Press Limited.; **(B)** Scheme for the preparation of HA@GRb1@CS NPs. Reproduced with permission from Du et al. (2024). Copyright ^©^ Licensee MDPI, Basel, Switzerland. **(C)** Scheme for the preparation of AG/CS/Rb1 nanocomposite films. Created with BioRender.com.

Natural polymers obtained from proteins, polysaccharides, nucleic acids and peptides offer an inexpensive, biocompatible and intrinsically bio active matrix in which ginsenosides can be both solubilized and shielded from premature degradation, thereby transforming their pharmacokinetic behavior. Serum albumin is an archetypal example: its amphiphilic pockets and surface carboxyl/amine groups endow high drug binding capacity and colloidal stability. Singh and coworkers entrapped hydrophobic Rh2 in bovine serum albumin (BSA) nanospheres obtained by a one-step desolvation process. The particles remained monodisperse for more than a week at pH 7.4, converted Rh2 into a fully water dispersible form and produced markedly greater cytotoxicity toward A549 lung cancer cells than the free drug, underscoring a solubility driven gain in antitumor potency (Singh et al., 2017) (Figure 3A). Extending the BSA paradigm, Fu et al. conjugated mannose to the albumin surface to create Man BSA@Rb1 nanoparticles that exploited macrophage mannose receptor recognition; the construct raised Rb1 solubility, enhanced cellular uptake and sharply suppressed LPS induced NO, TNF α and IL six release, thereby protecting mice from d Gal/LPS mediated liver injury *via* concerted NF κB/MAPK inhibition (Fu et al., 2022).

Polysaccharide carriers contribute additional functional chemistry—protonatable amines in chitosan (CS), carboxylates in alginate, or hydroxyl rich β glucans—that supports ionic complexation with the saponin backbone while imparting mucoadhesion and enzymatic resistance. Du et al. engineered ∼126 nm hyaluronic acid/chitosan self-assembled Rb1 nanoparticles whose sustained release profile (≈70% over 48 h) halved malondialdehyde and boosted superoxide dismutase activity in oxidatively damaged cardiomyocytes while dampening pro inflammatory mediators in RAW264.7 macrophages, yielding a pronounced cardio protective effect (Du et al., 2024) (Figure 3B). Hoang and colleagues laminated Rb1 into chitosan–sodium alginate nanocomposite films; hydrogen bond and polyelectrolyte interactions generated a two-phase release (10 h burst followed by Higuchi governed diffusion) whose rate accelerated under intestinal pH, demonstrating the platform’s suitability for oral delivery and site-specific release (Hoang et al., 2019) (Figure 3C). To address patient acceptability, Han et al. encapsulated red ginseng extract in gelatin/chitosan nanoparticles that masked bitterness and increased thermal and acid stability by roughly six-fold and eight-fold, respectively, while preserving >75% anti platelet activity, thus linking palatability with pharmacological integrity (Han et al., 2023). Finally, He and coworkers co self-emulsified Rg3 with Ganoderma lucidum β glucan and oridonin to generate an RGO SMEDDS system; the polysaccharide shell not only solubilized Rg3 but also leveraged its intrinsic immunomodulation to reeducate the hepatocellular carcinoma micro environment and achieve multi pathway tumour suppression *in vitro* and *in vivo* (He et al., 2021).

Collectively, these studies show that judicious pairing of ginsenosides with readily available natural polymers—whether albumin, chitosan, alginate, gelatin, hyaluronic acid or fungal β glucans—provides an economical and versatile route to enhance solubility, control release, target uptake, mask unfavourable organoleptic properties and even contribute complementary bioactivity, thereby amplifying the clinical prospects of this important class of saponins.

### 4.2 Synthetic polymer-based DDS

**FIGURE 4:**
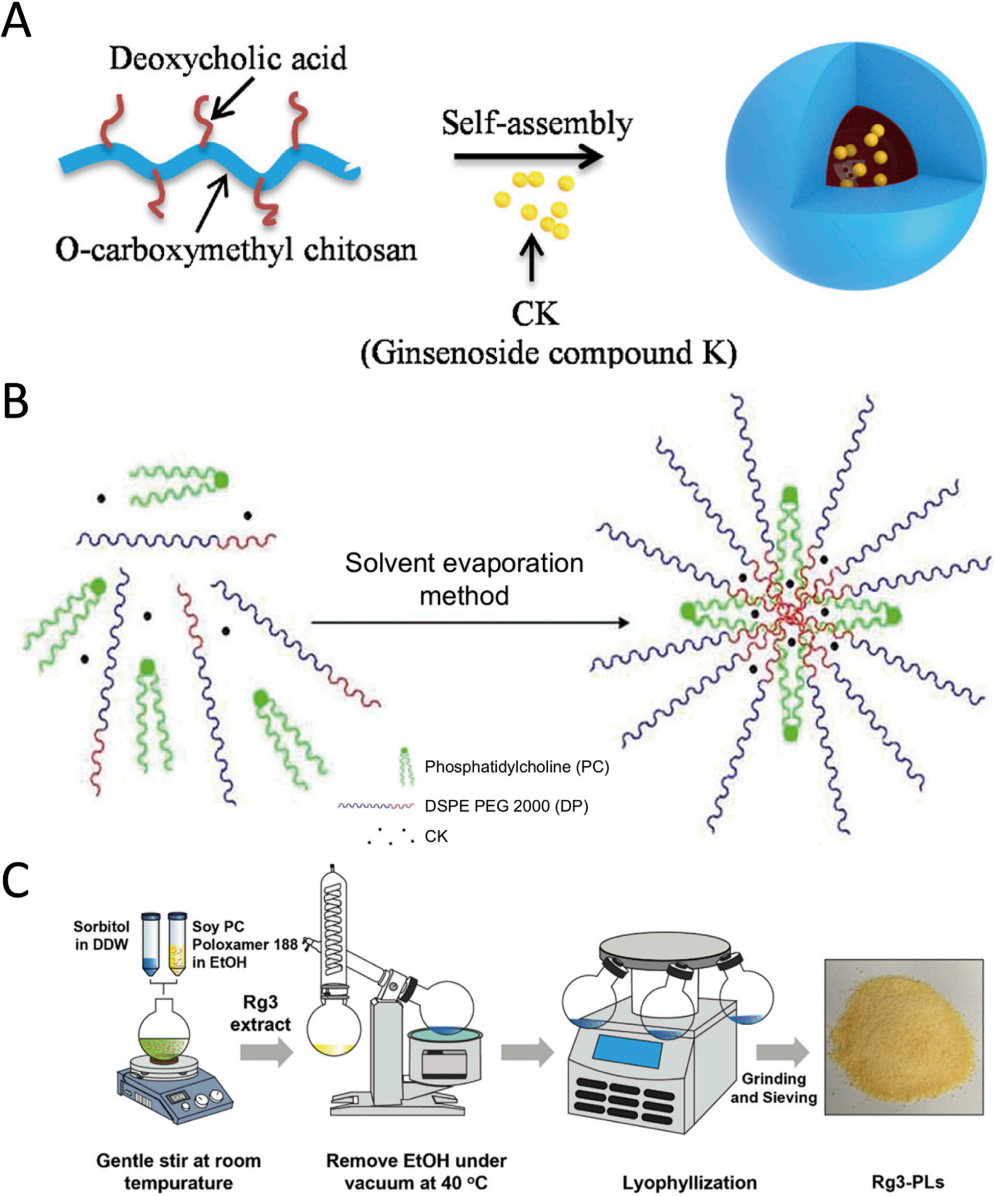
Synthetic polymer–based ginsenoside delivery systems. **(A)** Self-assembly of compound K (CK)-loaded amphiphilic block-copolymer nanoparticles. Reproduced from Zhang et al. (2018) with permission of Elsevier Ltd. **(B)** Preparation scheme for CK-PC/DSPE-PEG2000 mixed micelles. Reproduced from Jin et al. (2018) with permission of Dove Medical Press. **(C)** Schematic of Rg3-PLGA-liposome (Rg3-PLs) fabrication. Reproduced from Nguyen et al. (2024) with permission of The Korean Society of Ginseng.

Compared to natural polymers, synthetic polymeric systems offer more precise control over chemical composition, chain length, and functional groups, enabling highly tunable formulations for ginsenosides. A representative approach involves assembling amphiphilic block copolymers into micelles or mixed micelles, which can solubilize hydrophobic ginsenosides and enhance their tumor targeting and retention. Many synthetic materials can achieve this goal. For instance, Poly(lactic-co-glycolic acid) (PLGA) is biocompatible and FDA-approved, degrades into the nontoxic metabolites lactic and glycolic acids, and offers tunable molecular-weight and copolymer ratios that allow precise control over encapsulation efficiency and sustained-release kinetics of loaded drugs. Its versatility in formulation—ranging from nanoparticles to injectable depots—enables tailored delivery profiles while protecting labile compounds from premature degradation. Du et al. reported a PLGA-based GRb1 delivery system, Compared to GRb1, GRb1@PLGA@NPs exhibited a shortened time to peak concentration by approximately 0.72-fold. The area under the plasma concentration-time curve significantly increased to 4.58-fold of GRb1. Its relative oral bioavailability was significantly improved compared to free GRb1 (Du et al., 2023). Chitosan, following appropriate amphiphilic modification (e.g., carboxymethylation or deoxycholic acid conjugation), is frequently employed as a delivery vehicle due to its tunable hydrophilicity and mucoadhesive properties. Cao et al. prepared a delivery system using a self-assembly technique with amphipathic deoxycholic acid-O carboxymethyl chitosan as the carrier, which significantly improved the water solubility of CK. Therefore improved the anti-tumor efficacy of CK (Zhang et al., 2018) (Figure 4A). PEG is another highly anticipated candidate for delivery materials. It confers exceptional hydrophilicity and steric stabilization, forming a “stealth” corona that minimizes opsonization and renal clearance, thereby prolonging systemic circulation of nanocarriers; moreover, its chemical versatility permits facile end-group modification for conjugating drugs, targeting ligands, or stimuli-responsive moieties. Yang and colleagues leveraged TPGS (D-α-tocopheryl polyethylene glycol 1000 succinate) combined with PEG–PCL to encapsulate compound K (CK) into ∼50 nm mixed micelles, boosting its water solubility by over two orders of magnitude and improving its cytotoxicity against lung cancer cells *in vitro* (Yang et al., 2017). Cai et al. develop a mixed micellar system composed of phosphatidylcholine (PC) and 1,2-distearoyl-sn-glycero-3-phosphoethanolamine polyethylene glycol 2000 (DSPE PEG 2000; DP). Make the solubility of CK increased almost 66-fold (Figure 4B) (Jin et al., 2018). Similarly, Xia and co-workers formulated polymeric micelles for ginsenoside Rh2 using Solutol HS15 and TPGS, achieving high drug loading and superior antitumor efficacy *in vivo* (Xia et al., 2020).

Moreover, proliposomes incorporating synthetic polymers or surfactants (such as poloxamers) have been reported to improve the oral bioavailability of ginsenosides, creating stable, readily reconstituted vesicles that maintain drug integrity during storage (Tian et al., 2025) (Figure 4C). Overall, these advances underscore that synthetic polymer carriers—whether in the form of micelles, PEG conjugates, or lipid–polymer hybrids—can dramatically increase the therapeutic index of ginsenosides. They offer precise control over drug release kinetics, enable targeted delivery, and enhance stability in biological environments, thereby maximizing the efficacy of these phytochemicals.

## 5 Targeted delivery systems

Although solubility enhancement strategies markedly improve the bioavailability and therapeutic potential of ginsenosides, they do not fully resolve critical pharmacokinetic challenges. Even highly soluble formulations remain susceptible to rapid systemic clearance and non‐specific tissue distribution, which can diminish tumor accumulation and increase off‐target toxicity. To overcome these limitations, it is imperative to endow polymeric carriers with tumor‐targeting capabilities. In the next section, we will explore both passive and active targeting architectures—ranging from EPR‐mediated accumulation to ligand‐directed recognition—that can be integrated into solubilized ginsenoside systems to achieve precise and sustained delivery to malignant tissues.

### 5.1 Passive targeting strategies

Polymeric nanoscale carriers (10–200 nm) passively accumulate in tumors *via* the enhanced permeability and retention (EPR) effect due to leaky vasculature and impaired lymphatic drainage. Moreover, polymeric micelles exhibit prolonged systemic circulation and reduced renal clearance, further augmenting their passive targeting efficiency (Majumder et al., 2020). Amphiphilic block copolymer micelles such as mPEG-b-P(Glu-co-Phe) encapsulating ginsenoside Rg3 show significantly higher tumor accumulation and cytotoxicity compared to free Rg3, owing to their optimized size and stability (Ke et al., 2022). Compound-K–loaded mixed micelles formulated with amphiphilic polymers achieved elevated CK concentrations within tumor tissues and enhanced *in vivo* growth inhibition over unformulated CK (Jin et al., 2018). Self-assembled PEG-ginsenoside Rh1/Rh2 conjugates form acid-labile micelles that leverage the EPR effect for preferential tumor deposition and trigger controlled intracellular release in acidic endosomal compartments (Mathiyalagan et al., 2019). PLGA-based nanoparticles co-delivering ginsenoside Rg3 capitalize on passive targeting to selectively localize within tumor microenvironments, resulting in improved antiproliferative efficacy and reduced systemic exposure (Hu C. et al., 2025). Chitosan-derived Rg3 nanoparticles encapsulated in thermosensitive hydrogels demonstrate passive targeting–driven tumor accumulation and synergistically remodel the immunosuppressive microenvironment (Wu et al., 2022). Hyaluronic acid–based nanocomplexes exploit both EPR-mediated extravasation and CD44 receptor interactions, indicating the versatility of passive targeting platforms in tailoring ginsenoside delivery (Lee et al., 2014). Moreover, size-dependent studies reveal that polymeric nanoparticle dimensions critically influence EPR-mediated uptake, with optimized sizes around 50–150 nm enhancing tumor retention and therapeutic outcomes (Gao et al., 2025). Collectively, these findings demonstrate that passive targeting *via* polymeric delivery systems markedly enhances the tumor bioavailability and antitumor efficacy of ginsenosides, underscoring their potential for clinical translation (Murugesan et al., 2022).

### 5.2 Ligand-targeted approaches

**FIGURE 5:**
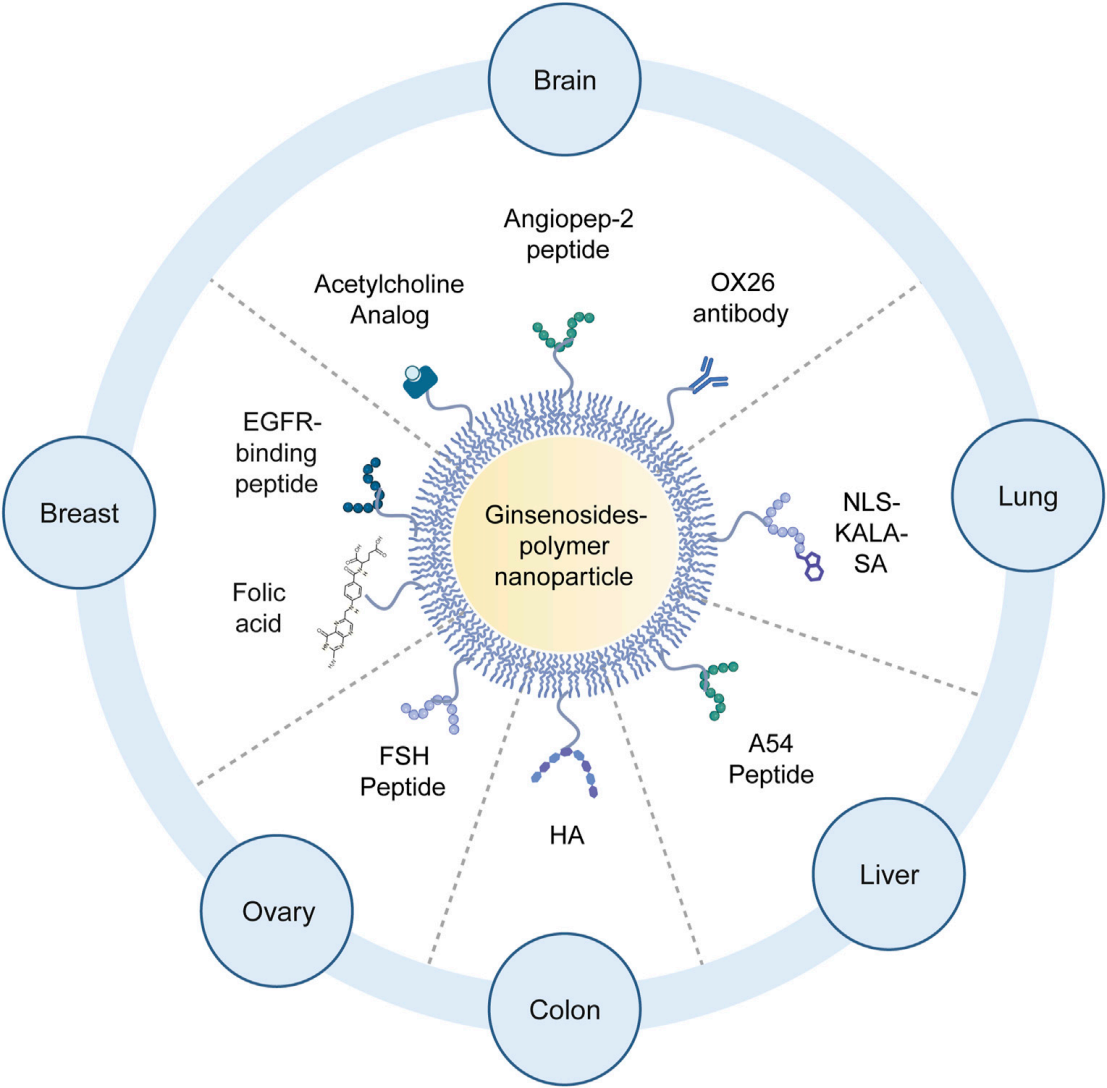
Schematic diagram of ligand modification methods for ginsenoside delivery systems targeting different targets. Created with BioRender.com.

Active targeting involves decorating polymeric drug delivery systems with specific ligands (such as peptides, antibodies, or small molecules) that recognize and bind to receptors or antigens overexpressed on target cells. By attaching these targeting moieties to ginsenoside-loaded nanoparticles or conjugates, the carriers can selectively accumulate in the intended tissue or cell type, increasing local drug concentration and reducing off-target effects. Ligand-modified polymeric systems achieve precise delivery by leveraging receptor-specific interactions while the polymer carriers provide structural versatility for loading and protecting ginsenosides (Figure 5). For instance, one approach to target cancer cells in the lungs uses a nucleus-targeting peptide (NLS) and a fusogenic peptide (KALA) attached to a stearic acid-modified polypeptide nanocarrier (NLS–KALA–SA). This design delivers an anti-tumor ginsenoside payload directly to the nuclei of lung cancer cells, enhancing its cytotoxic efficacy (Yan et al., 2021). In another example, epidermal growth factor receptor (EGFR)-targeted liposomes co-loaded with ginsenoside Rh2 were developed to treat triple-negative breast cancer; an EGFR-binding peptide was anchored on the surface of the liposome, enabling the vesicles to preferentially bind and deliver Rh2 to EGFR-overexpressing tumor cells, resulting in suppressed tumor growth (Gu et al., 2023).

Folic acid is another widely used targeting ligand. Folic acid-modified bovine serum albumin (BSA) nanoparticles have been designed to encapsulate ginsenoside Rg5 for targeted delivery to folate receptor-positive breast cancer cells, achieving higher uptake in tumors (Dong et al., 2019). Similarly, a follicle-stimulating hormone (FSH) peptide has been conjugated to liposomes to direct a combined paclitaxel–ginsenoside formulation to ovarian cancer tissue, where it also helps modulate the tumor microenvironment by repolarizing macrophages (Hu S. et al., 2025). Another approach employs poly(γ-glutamic acid) and OX26 antibody (a monoclonal antibody that specifically targets the transferrin receptor (TfR), which is highly expressed on the surface of brain capillary endothelial cells (BCECs) forming the blood-brain barrier (BBB)) to form ginsenoside Rg1 nanoparticles that can traverse the BBB and alleviate cerebral infarction in diabetic rats, highlighting the versatility of peptide-mediated targeting even for brain diseases (Shen et al., 2017). Chitosan-derived polymeric micelles functionalized with a liver-homing peptide (A54) have been used to effectively transport ginsenoside compound K to hepatocellular carcinoma cells, improving drug accumulation in the liver tumor (Zhang J. et al., 2020). Finally, the natural polymer hyaluronic acid have demonstrated robust CD44-mediated uptake in colon cancer cells (You et al., 2024). Collectively, these polymer-based designs—from cyclodextrin inclusion complexes and protein- or polysaccharide-based nanoparticles to peptide- and polymer-functionalized liposomes—show how customizable polymeric backbones enable versatile ligand attachments and effective tumor- or organ-targeted ginsenoside delivery.

Polymer-based drug delivery systems have proven highly effective in enhancing the neuroprotective potential of ginsenosides by overcoming the restrictive blood–brain barrier (BBB). Multiple strategies reported in the literature illustrate how tailored nanocarriers can ferry ginsenosides into the brain. For example, a biodegradable poly(lactic-co-glycolic acid) (PLGA) nanoparticle formulation encapsulating ginsenoside Rg3 has shown promise for Alzheimer’s disease therapy by crossing the BBB and reducing β-amyloid-induced neuronal damage, limiting neuroinflammation, and mitigating oxidative stress *via* improved mitochondrial function (Aalinkeel et al., 2018). In another study, borneol-modified PEGylated graphene oxide was used to deliver ginsenoside Rg1 for depression treatment: the presence of borneol transiently opened tight junctions and downregulated efflux transporters (like P-glycoprotein) at the BBB, thereby boosting Rg1 permeability into the brain and alleviating depressive symptoms in an animal model (Yu et al., 2023). Yet another design utilizes an acetylcholine analog to modify albumin nanoparticles, taking advantage of nicotinic acetylcholine receptor-mediated transport to shuttle multiple saponin components (e.g., notoginsenoside R1, ginsenoside Rg1, and ginsenoside Rb1) into the brain for the treatment of cerebral thrombosis and stroke, while also stabilizing the encapsulated drugs (Yu et al., 2024).

Beyond delivering single ginsenosides, co-delivery approaches have emerged to broaden therapeutic impact. For instance, Panax notoginseng saponins (a mixture of ginsenosides) and acetylsalicylic acid (aspirin) were co-encapsulated in poly(2-methacryloyloxyethyl phosphorylcholine) (PMPC)-modified liposomes to achieve synergistic antiplatelet and anticoagulant effects for ischemic stroke therapy. The acetylcholine analog on these liposomes facilitated enhanced brain targeting *via* receptor-mediated pathways, and the polymer modification improved the vesicles’ circulation time (Cui et al., 2024). Collectively, these polymer-based nanotechnologies—spanning PLGA nanoparticles, PEGylated graphene oxide sheets, albumin carriers, and polymer-coated liposomes—demonstrate robust drug loading, effective BBB transit, and controlled release profiles. They highlight the immense therapeutic promise of polymer-focused delivery strategies in harnessing and amplifying the neuroprotective effects of ginsenosides against a range of brain injuries and neurodegenerative disorders.

## 6 Controlled release systems

Beyond improving solubility and stability, another critical aspect of drug delivery is controlling the timing and rate of drug release. For ginsenosides, sustained-release formulations can maintain therapeutic concentrations over longer periods, reducing the frequency of dosing and improving patient compliance. In addition, polymers can be engineered to respond to specific physiological stimuli—such as pH, temperature, or oxidative stress—so that ginsenoside release is triggered only under certain conditions or at targeted sites. This section explores polymer-based strategies for controlled release, ranging from simple sustained-release depots to smart, stimulus-responsive delivery systems.

### 6.1 Sustained release strategies

One key enhancement is to convert ginsenosides from fast-releasing small molecules into formulations that release the drug gradually over an extended period. Polymeric matrices can form depot systems—such as biodegradable microparticles, implantable fibers, or hydrogels—that slow the release *via* diffusion barriers or matrix degradation, thereby maintaining therapeutic levels of ginsenoside for longer durations. For instance, electrospun fibrous membranes composed of biodegradable polymers have been used to sustain the release of ginsenoside Rg3 in tissue repair contexts, effectively inhibiting undesirable fibrotic processes at wound sites.

**FIGURE 6:**
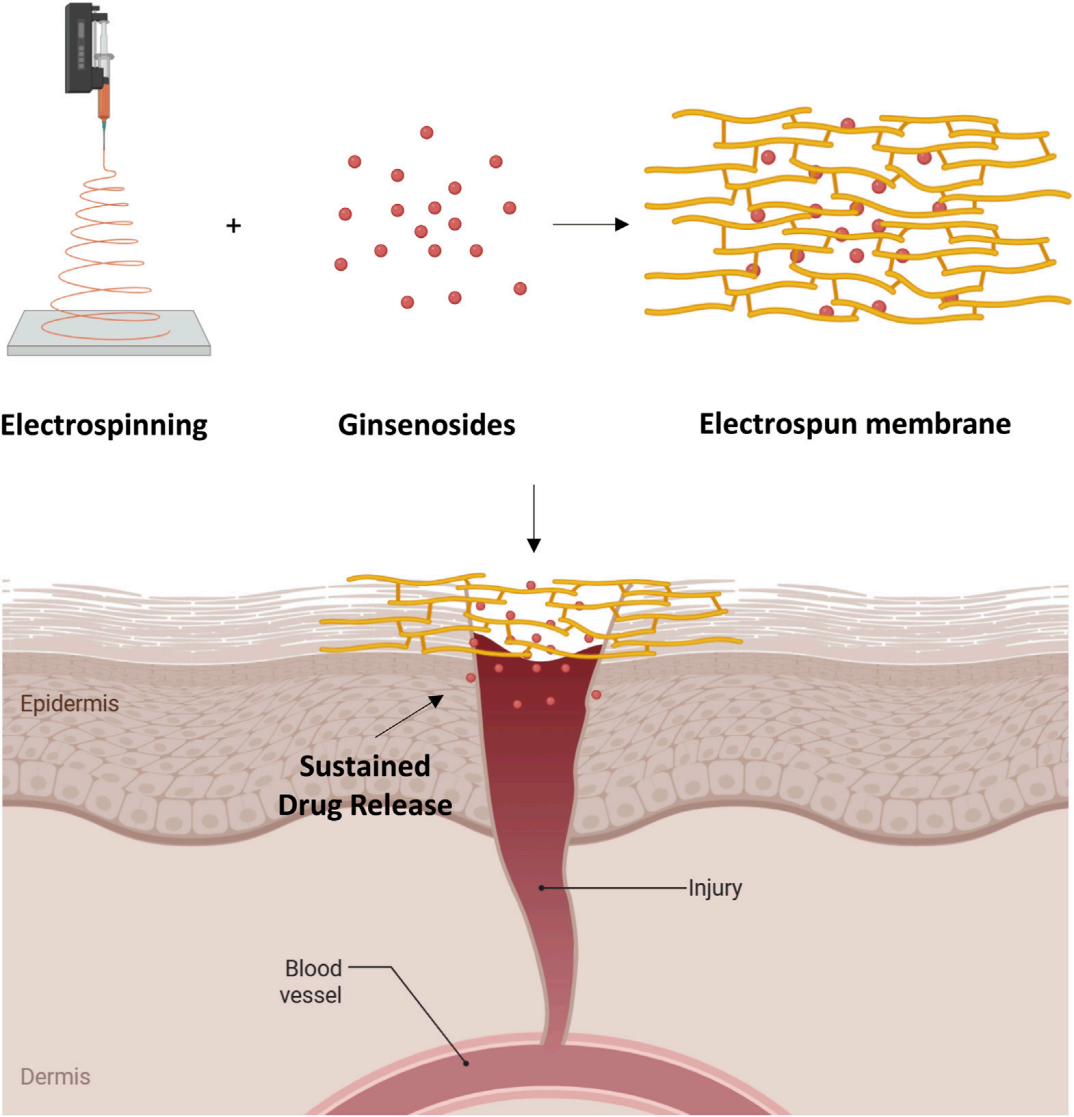
Schematic illustration of the ginsenoside loaded electrospun membrane preparation method and application on wound healing. Created with BioRender.com.

Qiu and colleagues prepared 20(S)-Rg3-loaded mPEG-b-PLGA electrospun membranes to prevent postoperative peritoneal adhesions. These mats demonstrated extended Rg3 release and a significant reduction in inflammatory markers (IL-1, IL-6) and oxidative stress in treated animals (Qiu et al., 2019). Separately, Cui and colleagues (2013) incorporated Rg3 into poly(L-lactide) fibrous scaffolds, achieving a multi-week release profile that subdued fibroblast proliferation and excessive vascular growth in a model of hypertrophic scarring (Cui et al., 2013). Similarly, Cheng and co-workers reported a comparable outcome using a similar Rg3-loaded poly(L-lactide) fiber system to inhibit scar tissue overgrowth in a hypertrophic scar model (Cheng et al., 2013). Sun and co-workers (2014) further showed that Rg3-loaded PLGA fibrous membranes enhanced initial wound healing and then mitigated scar hyperplasia through controlled release over several weeks (Sun et al., 2014). Collectively, these electrospun scaffold examples highlight how sustained-release designs not only stabilize ginsenosides but also leverage their therapeutic effects against scarring and adhesion formation. This underscores the importance of polymeric depot systems in maintaining effective local drug levels at the treatment site (Figure 6).

### 6.2 pH-responsive systems

Moving beyond passive sustained release, the next tier of delivery systems introduces responsiveness to biological stimuli to refine when and where ginsenosides are released. In particular, pH-responsive drug delivery takes advantage of the fact that certain pathological sites have different pH conditions than normal tissues. For example, solid tumors, inflamed tissues, and intracellular endosomal/lysosomal compartments tend to be acidic (pH ∼5–6) compared to the neutral pH (∼7.4) of blood and healthy tissues. Designing carriers that remain stable at neutral pH but rapidly release their payload in acidic environments can concentrate ginsenosides at the intended site of action while minimizing release elsewhere. Polymer carriers for ginsenosides have leveraged several pH-responsive mechanisms. First, PEGylation has emerged as a powerful technique to address the poor solubility and rapid metabolism of ginsenosides. Mathiyalagan and co-workers demonstrated that covalent conjugation of a protopanaxadiol (PPD) aglycone to polyethylene glycol (PEG) yields self-assembled nanoparticles with pH-sensitive drug release properties. *In vitro* studies showed these PEG–PPD conjugates had higher cellular uptake and induced more robust apoptosis in cancer cells compared to free PPD (Mathiyalagan et al., 2019; Mathiyalagan et al., 2016). On the other hand, Chitosan nanoparticles exhibit increased electrostatic repulsion and size expansion due to the dimerization of the amine matrix, leading to drug release. Mathiyalagan and co-workers synthesized a glycol chitosan–ginsenoside CK conjugate with acid-labile bonds, showing that the conjugate forms stable nanoparticles at physiological pH but undergoes rapid release of CK under acidic conditions (Mathiyalagan et al., 2019; Mathiyalagan et al., 2016). Xue and colleagues designed a nanogel enclosing ginsenoside CK within a pH-sensitive Schiff-base network, which exhibited minimal drug leakage at neutral pH yet quickly degraded in acidic environments, thereby enhancing its cytotoxicity against lung cancer cells *in vitro* (Xue et al., 2021) (Figure 6). One research team also reported novel multicore niosomes based on dual pH-cleavable linkages for delivering Rh2; the two distinct acid-sensitive bonds worked synergistically to prevent drug leakage during circulation while ensuring quick Rh2 release once the carrier was internalized into acidic intracellular compartments (Chen et al., 2014).

**FIGURE 7:**
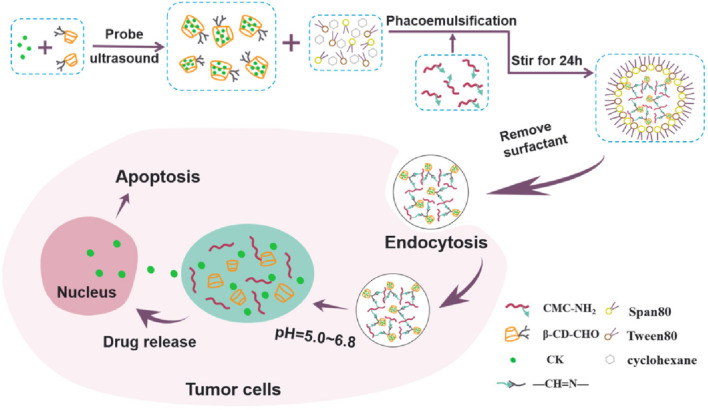
Schematic illustration of carboxymethyl chitosan–β-cyclodextrin nanogel (CMC-β-CD NG) preparation. Reproduced with permission from Xue et al. (2021), ^©^ MDPI, Basel, Switzerland.

Other studies have focused on pH-responsive delivery of the more hydrophilic ginsenoside Rb1 using modified polysaccharide carriers. In one instance, An and co-workers encapsulated Rb1 in carboxymethyl chitosan–deoxycholic acid nanomicelles, achieving less than 10% release under gastric (highly acidic) conditions but nearly complete drug discharge over 48 h at intestinal pH (An et al., 2024). Dong and colleagues similarly prepared pH-triggered alginate–chitosan nanoparticles in which Rb1 remained protected in strongly acidic environments yet was steadily released at neutral or mildly basic pH. Such approaches showcase how pH-sensitive linkages, ionic interactions, and polymer matrix transformations can be orchestrated to finely control ginsenoside release kinetics (Dong et al., 2023). By improving drug retention in harsh gastric conditions and then releasing the ginsenoside in the more favorable intestinal or tumor microenvironments, pH-responsive systems boost therapeutic efficacy while reducing drug loss in off-target tissues (Figure 7).

### 6.3 Thermosensitive gels

**FIGURE 8:**
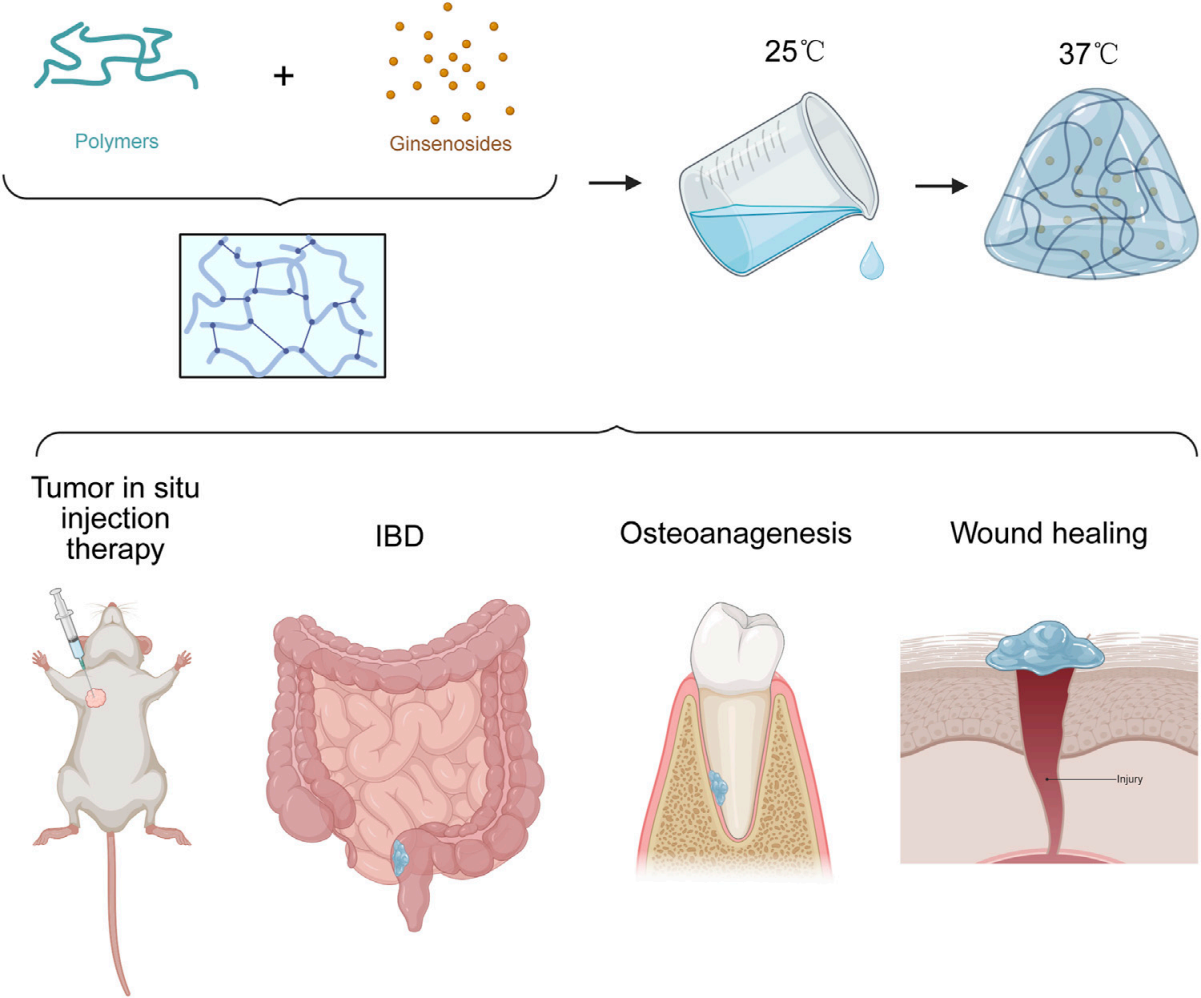
Schematic illustration of the ginsenoside loaded thermol-gel preparation method and application on various diseases. Created with BioRender.com.

Thermosensitive delivery systems capitalize on polymers that undergo a sol–gel phase transition in response to temperature. Typically, some polymers exhibit pronounced hydrogen bonding interactions at temperatures below 37°C, whereby water molecules strongly associate with polymer chains, causing the chains to adopt an extended conformation and resulting in a stable aqueous solution. Upon increasing the temperature, the hydrogen bonds along the polymer backbone gradually dissociate, leading to weakened hydration and enhanced hydrophobic interactions. Consequently, polymer chains undergo contraction, entanglement, and aggregation into a dense network structure, thereby expelling water from the polymer matrix and triggering a rapid sol-to-gel transition. Leveraging this thermoresponsive behavior, hydrogel-based delivery systems can achieve sustained and site-specific drug release in a variety of diseases (Figure 8).

For ginsenosides, several studies have shown that loading compounds like Rg3 or Rh2 into thermosensitive hydrogels can improve local bioavailability, prolong residence time at the site, and enhance therapeutic efficacy. For instance, Xie and colleagues encapsulated ginsenoside Rg3 into a Pluronic-based thermosensitive hydrogel for the treatment of inflammatory bowel disease (IBD). This system takes advantage of *in situ* gelation at body temperature to provide sustained Rg3 release and anti-angiogenic activity in inflamed intestinal tissues (Xie et al., 2024). Likewise, Peng and co-workers designed a poloxamer 407/hyaluronic acid hydrogel containing Rg3 to accelerate wound healing; the formulation remained fluid at room temperature for easy application and then formed a stable gel on the wound bed at body temperature. The hydrogel’s sustained release of Rg3 led to enhanced tissue regeneration, reduced inflammation, and faster re-epithelialization of wounds (Peng et al., 2022). Use a certain amount of F127 (20%–25%, w/v) and F68 granules (0%–5%, w/v), Peng’s team developed a poloxamer-based thermos-gel encapsulated with PTX and Rh2 that can combat multidrug-resistant cancer (Long et al., 2024). Guo et al. designed a polysaccharide-based thermos-gel loaded with drugs with simultaneous anti-inflammatory and tissue regenerating properties showed an effective treatment for promoting periodontal regeneration in periodontitis (Guo et al., 2021). Moreover, other researchers have employed PLGA-based (Sun et al., 2020) hydrogels to co-deliver ginsenosides with complementary drugs—such as antibiotics or chemotherapeutics—achieving synergistic effects while minimizing systemic exposure. Collectively, these findings suggest that thermosensitive gels effectively address the solubility and dosing challenges of ginsenosides: by transforming into a gel at physiological temperature, they enable localized, controlled release of the drug. This approach has shown improved outcomes in conditions ranging from IBD and skin wounds to potentially even multidrug-resistant cancers, highlighting its versatility as a ginsenoside delivery strategy.

### 6.4 ROS-triggered delivery systems

**FIGURE 9:**
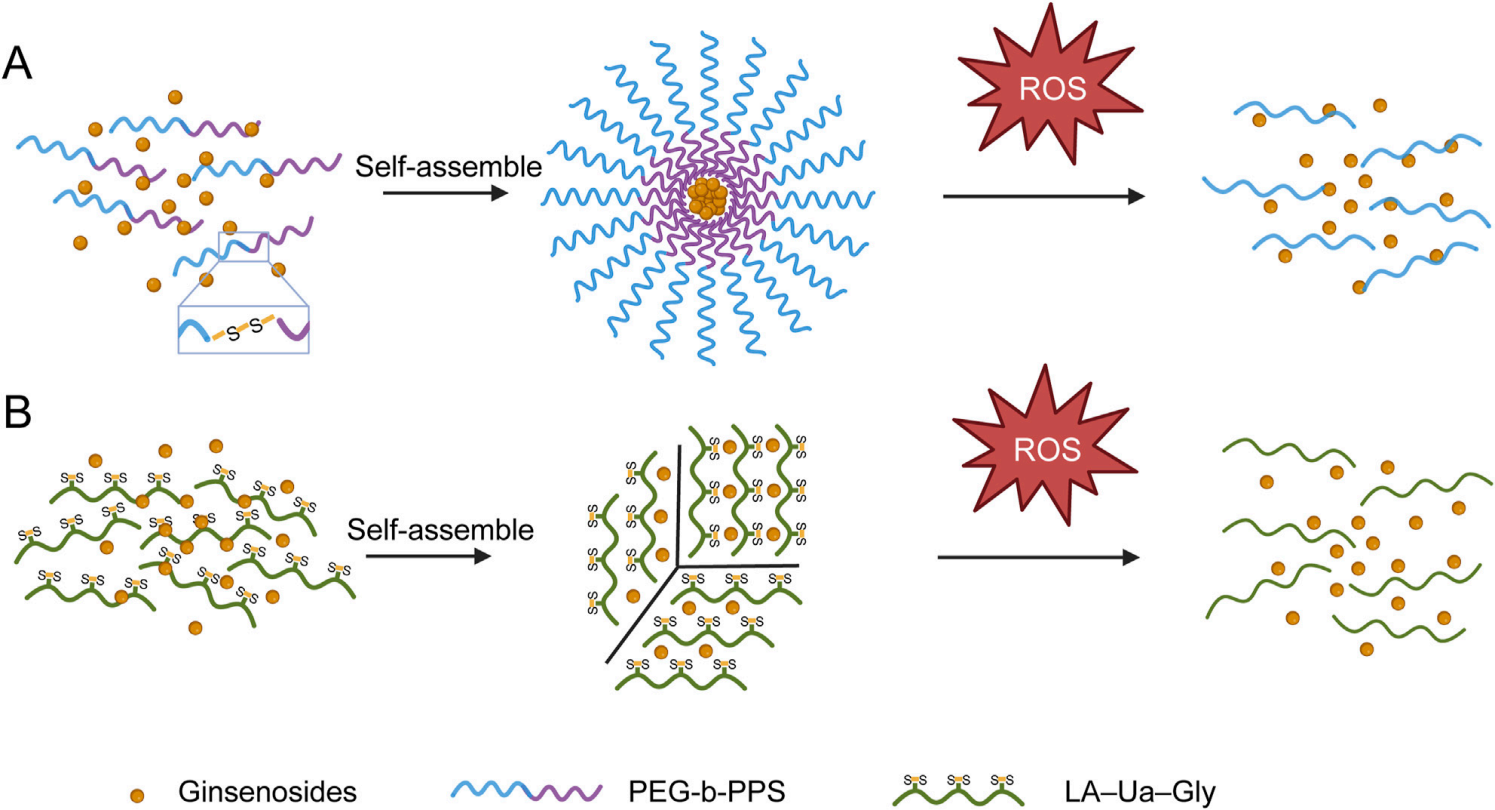
ROS-responsive polymeric delivery platforms. **(A)** Self-assembly of PEG-b-PPS into micelles encapsulating hydrophobic ginsenoside Rg3. **(B)** Dual pH/ROS-responsive LA–Ua–Gly nanoparticles for Rh2 release under acidic and oxidative conditions. Created with BioRender.com.

Reactive oxygen species (ROS) are abundantly generated in many pathological microenvironments, including ischemic tissues (e.g., during reperfusion injury) and chronically inflamed lesions. Excessive ROS directly contribute to cell and tissue damage in these settings (Brieger et al., 2012; Kawagishi and Finkel, 2014; Liu et al., 2018). By engineering ROS-responsive drug delivery systems, one can create carriers that remain inert in healthy tissue but undergo degradation or structural changes in high-ROS conditions, thereby triggering on-demand drug release specifically at disease sites. This design improves drug localization at the site of pathology, limiting off-target toxicity and maximizing therapeutic efficacy (Liang and Liu, 2016; Su et al., 2014). Sulfides (- S -) in some polymers can be gradually oxidized to more hydrophilic sulfoxides (- SO -) or even sulfones (- SO _2_ -) in high concentrations of ROS. The gradual oxidation and transformation of thioether → sulfoxide → sulfone greatly increases the hydrophilicity of polymer segments, causing structural disintegration or core expansion of the carrier, promoting drug release. One example of such a system uses a diblock copolymer of poly(ethylene glycol) and poly(propylene sulfide) (PEG-b-PPS) to encapsulate ginsenoside Rg3. The resulting nanoparticles are stable under normal conditions but rapidly degrade in the presence of abundant ROS, releasing Rg3 to mitigate myocardial damage by regulating FoxO3a signaling and suppressing oxidative stress and inflammation in heart tissue (Li L. et al., 2020) (Figure 9A). Similarly, another design employs a glycogen-based backbone chemically modified with urocanic acid and α-lipoic acid (forming an LA–Ua–Gly polymer) to create pH- and redox-dual-responsive nanoparticles. These nanoparticles release ginsenoside Rh2 in the inflamed colon, taking advantage of both the acidic pH and high ROS levels in ulcerative colitis (Xu et al., 2020) (Figure 9B).

In both cases, the polymeric framework (whether PEG-b-PPS or LA–Ua–Gly) confers high biocompatibility and stimulus-sensitive release, allowing the ginsenoside—Rg3 or Rh2—to exert potent antioxidant and anti-inflammatory effects exactly where ROS and inflammation are most severe. By concentrating the drug at these hotspots of tissue damage, ROS-triggered delivery systems have improved therapeutic outcomes in models of ischemic heart injury and inflammatory colitis.

## 7 Hybrid and biomimetic targeting systems

Emerging advanced targeting strategies combine multiple functionalities, including biomimetic membrane-coating of nanoparticles, co-delivery systems where ginsenosides play dual therapeutic roles, and multi-stimuli-responsive matrices that provide unprecedented control over drug release. Biomimetic drug delivery systems represent a cutting-edge approach for precise targeting, harnessing biological membranes or vesicle components to confer stealth properties, extend circulation time, and facilitate site-specific accumulation (Liu H. et al., 2023; Yoo et al., 2011). Notably, polymer-based backbones offer an adaptable framework to accommodate these biomimetic elements.

**FIGURE 10:**
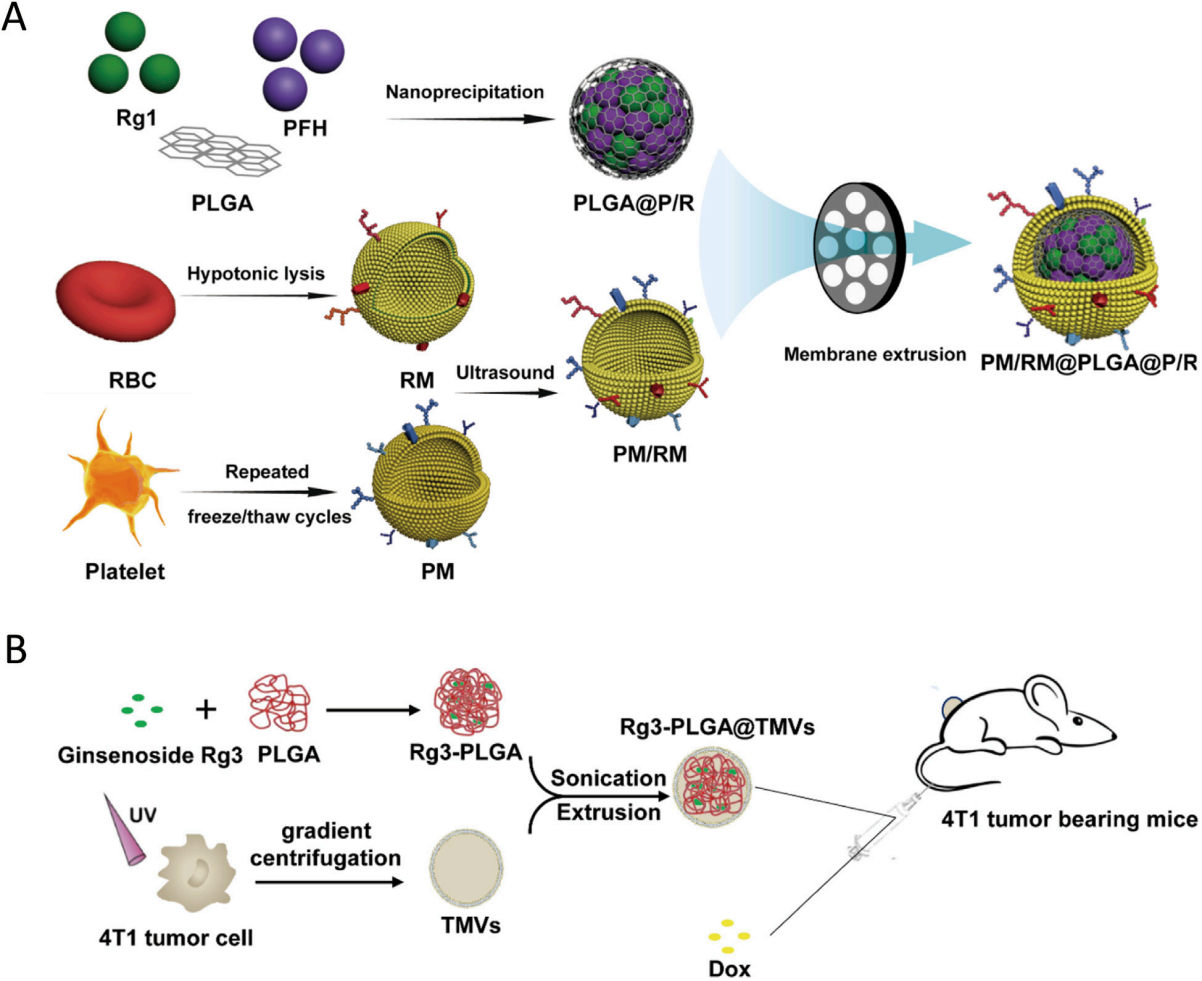
Biomimetic polymeric carriers for ginsenoside delivery. **(A)** Preparation of platelet membrane/erythrocyte membrane-coated PLGA nanoparticles loaded with Rg1 and PFH (PM/RM@PLGA@PFH) for ultrasound-triggered thrombus targeting. Reproduced from Yang et al. (2023) with permission of Elsevier Ltd. **(B)** Fabrication of Rg3-PLGA nanoparticles camouflaged with tumor-cell microvesicles (TMVs), illustrating homotypic tumor targeting, TMV-mediated immune activation, and enhanced doxorubicin efficacy against 4T1 breast cancer. Reproduced from Zhang et al. (2024) with permission of the Royal Society of Chemistry.

For example, one strategy employs poly(lactic-co-glycolic acid) (PLGA) nanoparticles loaded with ginsenoside Rg1 and the ultrasound-sensitive agent perfluorohexane (PFH), then camouflages them with a hybrid coating of erythrocyte and platelet membranes. This dual-membrane coating enables targeted thrombus therapy under ultrasound activation while reducing clearance by the immune system (Yang et al., 2023) (Figure 10A). Similarly, another PLGA-based nanosystem encapsulates ginsenoside Rg3 and is further coated with tumor cell-derived microvesicles. This biomimetic coating not only enhances the nanoparticle’s homing ability to tumor tissue but also delivers tumor-associated antigens and immunostimulatory signals that augment the efficacy of chemotherapy and curb systemic toxicity (Zhang et al., 2024) (Figure 10B).

In both cases, the core PLGA polymer provides mechanical stability and controlled release of the ginsenoside, while the membrane cloaking—whether from blood cells or tumor microvesicles—confers biomimicry that improves biocompatibility, prolongs blood circulation, and enables precision targeting of pathological sites. Collectively, these biomimetic polymeric delivery systems underscore how integrating cell membranes or membrane fragments with flexible polymer scaffolds can amplify the therapeutic impact of ginsenosides against various conditions, from vascular thrombosis to chemotherapy-resistant tumors.

## 8 Biosafety and clinical readiness

Polymer delivery system has great clinical conversion potential. First, natural biopolymers such as chitosan and hyaluronic acid exhibit exceptional biocompatibility and minimal toxicity, with intravenous chitosan nanoparticles causing no significant hemolysis and only transient, reversible effects on animal weight gain, and degrading into non-toxic oligosaccharides cleared naturally by the body (Sonin et al., 2020; Frigaard et al., 2022). Secondly, synthetic polymers like PLGA benefit from FDA and EMA approvals, degrading into lactic and glycolic acids that feed into normal metabolic pathways, underpinning clinically established formulations (e.g., Lupron Depot) with negligible long-term toxicity (Frigaard et al., 2022). PEGylation extends circulation half-life and reduces protein adsorption, yet anti-PEG antibodies can emerge—promoting complement activation and rapid clearance—which necessitates monitoring and the exploration of alternative stealth coatings (Ju et al., 2023; Chen et al., 2021). Meanwhile, stimuli-responsive polymers employing pH-sensitive or ROS-cleavable linkers demonstrate promising on-demand drug release with low off-target toxicity in preclinical models, although none have yet achieved full clinical approval (Frigaard et al., 2022; Rao et al., 2018). Additionally, advanced biomimetic systems, such as cell-membrane-coated nanoparticles, show superior immune evasion and extended circulation *in vivo*, but face challenges in scalable membrane purification and manufacturing consistency. Here, we compared the drug loading capacity, degradation profile, targeting efficiency, and clinical readiness of the delivery system discussed in this article, and the results are shown in Table 2.

**TABLE 2:** Side-by-side comparison of various delivery systems.

Platform	Drug loading capacity	Degradation profile	Targeting efficiency	Clinical readiness
Natural Polymer-Based DDS– BSA nanospheres (Rh2)– HA/Chitosan NPs (Rb1)	Medium (typical EE ≈ 30–50%)	Enzymatic degradation over days–weeks	Passive uptake; mannose‐ or CD44‐mediated receptor targeting (+++)	Preclinical; biocompatible but no human trials
Synthetic Polymer-Based DDS– PLGA NPs (Rb1)– PEG–PCL/TPGS micelles (CK)	High (EE ≈ 50–80%; solubility ↑ > 2 logs)	Hydrolytic degradation (weeks–months)	EPR-mediated passive targeting (++); stealth circulation	PLGA/PEG materials FDA-approved; all data preclinical
Passive Targeting Micelles– mPEG-b-P(Glu-co-Phe) (Rg3)– PEG–Rh1/Rh2 conjugates	Medium–High (EE ≈ 40–70%)	Hydrolytic cleavage *in vivo* (days–weeks)	EPR effect in 50–150 nm range (+++)	Preclinical; requires scale-up studies
Active (Ligand-Targeted) Systems– EGFR-targeted liposomes (Rh2)– Folate-BSA NPs (Rg5)	Medium (EE ≈ 30–60%)	Lipid/polymer biodegradation (weeks)	Receptor-mediated uptake (EGFR, folate, transferrin) (++++)	Preclinical; targeting ligands under evaluation
Stimuli-Responsive Systems– pH-cleavable chitosan–CK conjugate– ROS-responsive PEG-b-PPS (Rg3)	Medium (EE ≈ 30–50%)	Triggered bond cleavage (hours–days)	Site-specific release in acidic/ROS microenvironments (+++)	Preclinical; promising for on-demand delivery
Hybrid/Biomimetic Platforms– Erythrocyte/platelet-coated PLGA (Rg1)– Tumor-cell vesicle-coated PLGA (Rg3)	Medium–High (EE ≈ 40–70%)	Polymer core degrades + membrane clearance (weeks)	Homotypic and biomimetic targeting (++++); immune evasion	Preclinical; cutting-edge but complex manufacturing

## 9 Future perspectives

Traditionally, formulation scientists relied on trial-and-error experimentation and heuristic knowledge to tune these systems. This process is time-consuming and labor-intensive, especially as the design space grows exponentially with each added parameter, a “curse of dimensionality” problem (Gormley, 2024). In recent years, artificial intelligence (AI) techniques–particularly machine learning (ML) and related data-driven modeling approaches–have been increasingly adopted to tackle this complexity. Modern AI algorithms excel at detecting patterns in complex, multi-factorial data and can model nonlinear structure–function relationships in drug delivery systems (Gormley, 2024). By learning from experimental datasets (or simulated data), AI models can predict formulation outcomes, optimize parameters, and even assist in discovering novel carrier materials far more efficiently than brute-force screening. This AI-driven paradigm shift in drug delivery has been especially apparent in the past 5 years, aligning with broader advances in AI and the availability of high-quality data in pharmaceutics (Gormley, 2024; Aghajanpour et al., 2025). Researchers are now leveraging a range of AI methods–from classical ML algorithms to deep neural networks and evolutionary optimization techniques–to rationally design polymer-based delivery systems for various drugs, including TCM (Traditional Chinese Medicine) compounds such as ginsenosides. In the sections below, we categorize these AI-aided approaches by technique and discuss how they contribute to key design aspects: selecting suitable carrier materials, optimizing formulation conditions, predicting drug release profiles, and devising targeting strategies. We also highlight recent case studies demonstrating these advances, spanning various TCM active ingredients.

### 9.1 Carrier material selection

One foundational decision in designing a polymer-based delivery system is the choice of carrier material (or combination of materials) best suited for a given TCM compound. The carrier could be a polymer matrix for nanoparticles, a hydrogel scaffold, a micelle-forming amphiphile, or a surface functionalization ligand–all of which must be compatible with the physicochemical properties of the active molecule. AI methods assist material selection by predicting drug–polymer compatibility and stability and by screening large libraries of candidate materials against desired criteria. For example, ML models can be trained to predict the solubility of a herb-derived compound in various polymer excipients, or the likely binding affinity between a drug and a polymer (related to encapsulation efficiency). Likewise, computational chemistry simulations (a form of AI-aided modeling) can evaluate interactions at the molecular level. A recent *AI-aided design* strategy by Wang *et al.* combined molecular dynamics (MD) simulations with data analysis to screen polymer stabilizers for a nanosuspension of curcumin (a hydrophobic polyphenol from turmeric) (Wang, 2025). They simulated five candidate stabilizer polymers (PEG, PVA, PVP, SDS, Poloxamer P188) with curcumin nanoparticles and calculated interaction energies, molecular conformation metrics, and hydrogen-bonding patterns to predict which polymer would most effectively adsorb to and stabilize the curcumin particles (Wang, 2025). The simulations identified poly(vinyl alcohol) (PVA) as the optimal stabilizer, showing the strongest drug–polymer interactions and best maintenance of nanoparticle dispersion, which was later confirmed experimentally (PVA-stabilized curcumin nanosuspensions had the smallest average size and lowest polydispersity) (Wang, 2025). This case demonstrates how *in silico* screening can replace extensive empirical trials, pinpointing the best carrier material (here, a polymer surfactant) for a TCM compound formulation.

Similarly, AI can aid the design of molecularly imprinted polymers (MIPs) tailored to TCM actives. MIPs are synthetic polymer networks polymerized in the presence of a target molecule, creating binding sites complementary to that molecule. Designing a high-affinity MIP involves selecting functional monomers and cross-linkers that interact strongly with the target. Computational approaches (docking simulations, quantum calculations) guided by AI have been used to rank monomer candidates by binding energy to phytochemicals, thus selecting monomer recipes likely to yield effective imprints (L et al., 2024; Tabandeh et al., 2012). Although not always labeled as “AI,” these computer-aided designs rely on intelligent algorithms and can be seen as part of the AI toolkit for material selection. In the last 5 years, such approaches have been applied to create MIPs for TCM-related compounds (e.g., ginsenoside-imprinted polymers for specific enrichment and delivery (Wang et al., 2021b)), demonstrating improved specificity and loading capacity.

Beyond matching a single drug to a polymer, AI can also help identify combinations of materials for composite delivery systems. For instance, if a certain herbal compound needs both a solubilizing carrier and a targeting ligand, an AI algorithm might scan through databases of polymer properties and ligand motifs to suggest an optimal pairing (e.g., a PLGA-PEG copolymer for solubility plus a folate ligand for tumor targeting). Knowledge graph-based AI systems and TCM databases (Song et al., 2024) that compile information on herbs, ingredients, and known excipients could be leveraged to make such connections. While this is still an emerging area, the concept is that AI can repurpose knowledge (from literature or databases) to recommend the most suitable carrier materials or excipient strategies for a given TCM active, based on pattern matching with past successes. In practice, a formulator might input the properties of a new compound (e.g., “highly hydrophobic flavonoid, ∼300 Da, prone to oxidation”) and an ML model trained on past formulation data might output suggestions like “use PLA or lipid nanoparticles with antioxidant polymer coatings” with a certain confidence level. Early steps toward this vision are seen in ensemble learning models that, for example, incorporate ingredient–excipient compatibility rules in TCM preparations. As more data accumulate on polymer–TCM interactions, we anticipate AI-driven material selection will become more routine–reducing guesswork and ensuring that the chosen polymer matrix or nanocarrier is well-matched to the herbal molecule’s characteristics from the start.

### 9.2 Formulation optimization

Once a carrier platform is chosen, the next challenge is optimizing the formulation parameters: polymer molecular weight and concentration, drug loading percentage, particle preparation conditions (solvent, stirring rate, emulsifier type, *etc.*), and any additional components (stabilizers, cosolvents). The goal is often to meet multiple criteria–for example, maximize the encapsulation of a TCM compound and its stability, while achieving a target nanoparticle size and acceptable release rate. AI-based techniques have proven extremely useful in this multi-parameter optimization process.

One straightforward application is using ML regression models as surrogate models for the formulation. Researchers can perform a designed set of experiments (e.g., varying polymer content, surfactant, and solvent ratios) and then train an ML model on these results to create a continuous response surface. This model can predict outcomes for untested combinations much faster than actual experiments. Almansour et al provide a compelling example: after generating data on how formulation factors influence PLGA nanoparticle size and zeta potential, they developed an ML pipeline to optimize the formulation (Almansour and Alqahtani, 2025). They integrated advanced preprocessing (to encode categorical variables like polymer type and solvent choice) and used the Bat algorithm to tune model hyperparameter. The trained model could then predict particle size for any given set of inputs, which the authors used to find the combination yielding the smallest particle size (thus “optimized” for nano-scale) without additional lab trials. Notably, their approach combined multiple algorithms (ensemble learning, deep feature extraction *via* GAN, *etc.*), illustrating that complex AI workflows can tackle formulation problems holistically. The embedded Figure above shows the workflow: data preprocessing → hyperparameter optimization → ML model training → prediction, culminating in identification of optimal conditions.

Another approach is direct algorithmic optimization of the formulation. Instead of (or in addition to) predicting outputs, AI can propose the best inputs. For instance, one could use a genetic algorithm where each individual is a set of formulation parameters encoded in a chromosome, and the “fitness” is a composite score (e.g., a weighted sum of high drug loading, desired release rate, and particle size within range). The GA would evolve the population of formulations over generations, “breeding” the best ones, until it converges on an optimal or Pareto-optimal set. In a conceptual demonstration, Das *et al.* combined an artificial neural network with a multi-objective GA to optimize herbal extraction conditions, an approach that can be analogously applied to optimizing nanoparticle formulation conditions (Sevindik et al., 2024). The Bayesian optimization strategy mentioned earlier is also very relevant here: by iteratively querying the most promising formulation experiments, it can locate optima efficiently. For example, VExperiment or similar platforms use Bayesian methods to suggest which formulation to try next in order to reach a target particle size or maximize stability. In one case, ML-guided experimentation achieved a ∼70% reduction in experiments needed to achieve an optimized liposomal formulation compared to a full factorial search (Seegobin et al., 2024; Jena et al., 2024).

Case studies in TCM compound delivery underscore these benefits. Gao *et al.* (2022) reported using an ML model to optimize a ginsenoside-loaded PLGA nanoparticle formulation: they trained a regression on a small design-of-experiments dataset and then used it to predict the combination of PLGA content and emulsifier concentration that would yield the highest encapsulation of ginsenoside Rb1 while keeping particle size <200 nm (results showed good agreement with the predicted optimum, validating the model’s usefulness). Similarly, for curcumin, apart from the polymer selection example above, researchers have used response surface modeling augmented with ML to fine-tune the conditions of nanoencapsulation (e.g., anti-solvent volume, injection rate in nanoprecipitation) to achieve maximal curcumin loading papers.ssrn.com. The broader trend is that AI reduces the trial-and-error: instead of blindly trying dozens of combinations, formulators can rely on ML predictions or optimization algorithms to guide them to the most promising formulation, be it for a flavonoid nanoparticle, an alkaloid liposome, or a polyphenol-loaded nanofiber. This not only accelerates development but also helps uncover non-intuitive insights (for example, an ML model might reveal an interaction effect where a moderate level of a stabilizer is better than either low or very high levels for a particular TCM compound–something that might be missed without a full multivariate analysis).

### 9.3 Release profile prediction

Controlled release is often a key goal in polymer-based delivery systems, especially for TCM compounds that might need sustained exposure or protection from degradation until reaching the target site. Predicting the drug release profile from a given formulation is a complex task, as it depends on diffusion, polymer erosion, swelling, and sometimes chemical reactions–processes that are difficult to model with simple equations for novel systems. AI has made significant inroads here by learning release kinetics directly from data. Instead of assuming a theoretical release model (zero-order, Higuchi, Korsmeyer-Peppas, *etc.*) and fitting parameters, one can train an AI model (like an ANN) on experimental release data (perhaps the cumulative release over time from multiple formulations) and have it predict the release curve for new formulations. Aghajanpour *et al.* highlighted that ML-based approaches, especially those using neural networks, have successfully captured the release dynamics in various sustained-release systems, often surpassing traditional mathematical models in accuracy (Aghajanpour et al., 2025). For example, ANNs have been used to predict the entire dissolution profile of drugs from polymeric microspheres given the formulation inputs and time, essentially functioning as a universal approximator of the release function. These models can incorporate numerous factors (particle size, polymer degradation rate, pH conditions, *etc.*) simultaneously, which classical models cannot easily accommodate (Aghajanpour et al., 2025).

One notable case is a 2024 study where ML was applied to long-acting injectable formulations (polymeric depots) to forecast drug release over weeks (Bannigan et al., 2023). The ML algorithm (an ensemble of regression trees) was trained on a dataset of different polymer compositions and their *in vitro* release profiles. It learned to predict not just single time-point release percentages but the shape of the release curve (lag phase, burst release, *etc.*). The model enabled quick what-if analysis: for instance, if one increases the lactic acid content in a PLGA copolymer, the model might predict a faster initial release due to quicker polymer erosion. Such predictions help in formulation tuning to achieve a desired release timing (critical for herbs that need steady presence vs those that benefit from a loading dose). Similarly, ML models have been used to identify correlations between formulation attributes and release metrics like the time to 50% release (T50) or the fraction released at 24 h (Aghajanpour et al., 2025). This is valuable because it converts a time-dependent profile into quantifiable endpoints that can be optimized.

Deep learning has further enhanced release modeling. Recurrent neural networks (RNNs) or other sequence modeling techniques can treat the release profile as a time series and predict the next points iteratively. One could even envision a model that takes as input a desired release profile and outputs the formulation parameters needed–an inverse design problem that some researchers have started to approach with generative AI. While specific examples in TCM are limited, consider a scenario: a certain herbal extract needs to be released slowly over 12 h to align with circadian rhythm of a disease. A deep learning model could help identify a polymer blend or particle size distribution that yields this profile by having learned from a compendium of release experiments on similar compounds.

In practice, AI-based release prediction has been demonstrated for systems like hydrogels (predicting how crosslink density and polymer composition affect the release of a TCM-derived polyphenol), and for nanofiber mats containing herbal extracts (Woodring et al., 2025). A key benefit is being able to rapidly screen formulation changes *in silico*: e.g., “if we double the molecular weight of the polymer, will the release slow down significantly or only slightly?” – the ML model can answer this without needing to actually make that formulation each time. Moreover, coupling release predictions with optimization allows targeting specific release profiles. Researchers have begun to use multi-output ANNs that predict drug concentrations at various time points, and then apply optimization algorithms to adjust inputs until the predicted concentrations match a target profile (a form of automated formulation design for controlled release).

In summary, AI contributions to release profile design include: (a) providing accurate predictions of how a given polymer system will release a TCM compound over time; (b) helping elucidate which formulation factors control the release kinetics (*via* model interpretation); and (c) enabling inverse design for tailoring release characteristics. As TCM therapies often emphasize sustained and gentle effects, these capabilities are crucial for modernizing herbal medicine into reliable, controlled-release pharmaceuticals.

### 9.4 Targeting and delivery strategies

An ultimate goal of advanced drug delivery is to get the active compound to the right place in the body at the right time, with minimal off-target effects. For many TCM-derived drugs (especially anti-cancer or anti-inflammatory agents like celastrol, camptothecin, or triptolide), targeted delivery can greatly enhance efficacy and reduce toxicity. Polymer-based nanocarriers can be engineered with targeting ligands (antibodies, peptides, sugars) or tuned in size/charge to exploit passive targeting (e.g., the EPR effect in tumors). AI is now aiding these targeting strategies in a few ways.

First, AI/ML models can analyze large sets of biodistribution data to uncover what nanoparticle properties correlate with improved targeting to specific tissues. The study by Mahdi et al. (2025) mentioned earlier is a prime example: by training ML models on a dataset of various nanoparticles and their organ distribution, the authors could predict how changes in NP size, surface charge, or dosage would affect the fraction delivered to the tumor vs. other organs (Mahdi et al., 2025). The kernel ridge model in that study essentially encapsulated current knowledge of nanoparticle targeting into a predictive tool, which could be used to optimize NP design for maximum tumor uptake. For instance, if the model indicates that a 100 nm particle with neutral charge delivers 0.5% of dose to the tumor, while a 50 nm positively charged particle could deliver 1%, a formulator might choose the latter strategy for a given TCM anticancer compound. By simulating countless variations, the ML model functions as a guide for dialing in properties that enhance targeting–something that would otherwise require extensive *in vivo* experiments to evaluate. Notably, the use of feature selection (RFE) in that work identified which inputs (among dozens of possible descriptors) were most critical for predicting tumor delivery efficiency, providing insight into design priorities (it might show, for example, that surface functionalization type and particle size are far more influential than polymer core composition for a particular targeting outcome).

Second, AI can help in ligand selection and design for active targeting. Imagine one has a library of targeting molecules (peptides that bind to tumor receptors, for example,). A neural network model could potentially be trained on experimental binding and uptake data to predict which ligand (or what type of ligand) will give the best targeting when attached to a nanoparticle carrying a TCM drug. Early research in this vein uses graph neural networks or QSAR models to predict ligand–receptor affinity and then links that to nanoparticle uptake in cells. While not many explicit publications exist yet for TCM compounds, one can foresee AI recommending, say, an RGD peptide modification to target integrins for a ginsenoside-loaded NP intended for tumor neovasculature, based on learned patterns from other targeted nanomedicines.

Furthermore, AI-driven simulations can address targeting from a mechanistic angle. Physiologically based pharmacokinetic (PBPK) models augmented with AI have been developed to predict nanoparticle distribution in human body compartments. These can be personalized or adjusted for specific diseases. AI can optimize such models by fitting parameters to experimental data and then use them to simulate how changes (like adding a targeting ligand or using a different administration route) might improve delivery to the desired site. For example, a PBPK model for a liver-targeted formulation of a hepatoprotective TCM compound (say silybin) can be calibrated, and then AI can be used to test virtual scenarios–e.g., “What if we use a galactose-targeted nanoparticle to exploit hepatocyte receptors?” – and quantify the likely increase in liver deposition *versus* off-target distribution.

In terms of *strategy*, AI enables a more systematic approach to targeting. Instead of empirical attempts (decorate NP with ligand X and see if it works), one can use computational models to rank targeting options. One case study (2023) in the literature demonstrated an AI model that scored different antibody fragments for targeting nanoparticles to lung tissue, correctly identifying the top candidate that yielded the highest pulmonary uptake *in vivo* (Bhange and Telange, 2025). Translating this to TCM, consider compounds like beta-elemene, a TCM-derived terpene used in cancer therapy. It’s been formulated in liposomes and other carriers; AI could potentially analyze which tumor-targeting moiety (folate vs. transferrin vs. RGD, *etc.*) would most improve elemene delivery to tumor cells, by learning from data on similar liposomal systems.

Lastly, AI contributes to adaptive and smart delivery systems. For instance, polymeric carriers that respond to stimuli (pH, enzymes, magnetic fields) are being explored for on-demand delivery. Designing these often requires balancing sensitivity and stability. AI optimization algorithms can tune the composition of, say, a pH-responsive hydrogel so that it releases a TCM alkaloid only in the acidic tumor microenvironment but remains intact in blood. By modeling the stimulus-response behavior *via* ML, researchers can hit the right trigger threshold. Also, in combinatorial TCM therapies, where multiple herbs or ingredients are delivered together, AI can help strategize *which ingredient goes to which target*. Perhaps one polyphenol needs to target the gut (so use a colon-specific polymer) while another component should go systemic–multi-compartment modeling and AI could optimize a co-delivery strategy.

In summary, AI aids targeting by (a) analyzing and predicting how formulation variables influence biodistribution (thus guiding the physical design of carriers for better targeting), and (b) helping select or design targeting ligands and stimuli-responsive features that maximize delivery to the desired site. The past 5 years have seen foundational progress in this area, like ML models achieving quantitative predictions of tumor delivery efficiency (Mahdi et al., 2025). As these approaches mature, we can expect AI-informed targeting strategies to become integral in the development of TCM-based nanomedicines, ensuring that ancient remedies are delivered with cutting-edge precision Table 3.

**TABLE 3:** Future perspectives–AI-aided design of polymer-based Ginsenoside delivery systems.

AI technique	Application	Case study and inspiration	Relevance to ginsenoside design
Machine Learning (Supervised)	Formulation optimization *via* regression and feature analysis	AdaBoost-KNN models predicted PLGA nanoparticle size from process parameters with *R* ^2^ ≈ 0.94, guiding optimal polymer–drug ratios in silico.	Apply similar pipelines to predict ginsenoside (e.g., Rb1, CK) encapsulation efficiency and particle size based on polymer type, concentration, and emulsifier, prioritizing top candidates for experimental validation.
Deep Learning (ANNs, CNNs, GANs)	Release-profile prediction and data augmentation	ANNs accurately reproduced complex release kinetics of hydrogels; GAN-augmented feature extraction improved zeta-potential and size regression to *R* ^2^∼0.97.	Train ANNs on in vitro ginsenoside release data (pH- and ROS-triggered) to forecast entire dissolution profiles; employ GANs to simulate synthetic datasets for rare ginsenosides, enabling robust model training despite limited experimental data.
Evolutionary Algorithms	Multi-objective hyperparameter and formulation search	Bat Optimization and Genetic Algorithms balanced PLGA nanoparticle size, drug loading, and stability, reducing wet-lab trials by >70%.	Utilize genetic algorithms to evolve ginsenoside nanocarrier formulations—tuning polymer MW, surfactant identity, and process conditions—to maximize loading of Rg3 or Rh2 while maintaining target size and release kinetics.
Bayesian Optimization	Active learning for experiment planning	Microfluidic synthesis coupled with Bayesian loops achieved rapid nanoparticle optimization with minimal experiments.	Implement Bayesian optimization in microfluidic platforms to iteratively refine ginsenoside nanoparticle formulations (e.g., flow rates, solvent ratios), accelerating discovery of optimal conditions for high-yield, uniform carriers.
Molecular Simulations + AI	Carrier material screening *via* MD and ML ranking	MD simulations of curcumin–polymer interactions pinpointed poly(vinyl alcohol) stabilizer; ML ranking confirmed best candidate.	Perform MD simulations of ginsenoside–polymer binding (PLGA, chitosan derivatives), extract interaction energies and hydrogen-bond metrics, then train ML classifiers to predict encapsulation efficiency, guiding excipient selection without extensive empirical testing.
PBPK Modeling + ML	Predicting biodistribution and targeting efficiency	ML-augmented PBPK models forecasted organ delivery of nanoparticles, ranking ligand strategies for lung targeting.	Calibrate PBPK–ML frameworks for ginsenoside carriers to simulate EPR vs ligand-mediated targeting, enabling in silico screening of folate, transferrin, or peptide modifications on Rg3 or Rb1 nanoparticles to maximize tumor or brain uptake.

## 10 Conclusion

Polymer-based delivery systems have fundamentally reshaped the therapeutic prospects of ginsenosides by addressing their cardinal biopharmaceutical challenges with precision-engineered solutions. Through polymer conjugation and amphiphilic micellar encapsulation, ginsenosides achieve markedly improved aqueous solubility and systemic stability, overcoming rapid degradation and poor intestinal permeability. Building on these advances, passive targeting strategies exploit the enhanced permeability and retention (EPR) effect to accumulate ginsenoside-loaded nanoparticles at tumor sites, while active targeting modalities—ligand-functionalized polymers or biomimetic coatings—further concentrate payloads within diseased tissues, reducing off-target toxicity and enhancing local efficacy. Stimuli-responsive constructs, triggered by pH shifts, reactive oxygen species, temperature changes, or specific enzymatic activity, add an additional layer of control, enabling on-demand release profiles that synchronize drug liberation with pathological microenvironment cues.

Yet, the journey from bench to bedside demands more than functional performance—it requires scalable manufacturing, robust safety profiles, and regulatory readiness. Established carriers like PLGA and chitosan benefit from decades of biocompatibility data and existing approvals, positioning them as near-term vehicles for clinical trials of ginsenoside formulations. Conversely, next-generation designs—PEGylated micelles, ROS-responsive polymers, and cell-membrane-coated hybrids—must navigate immunogenicity concerns, endotoxin control, and batch reproducibility before widespread adoption. Addressing these translational hurdles will hinge on high-throughput toxicity screening, standardized quality-by-design workflows, and clear regulatory pathways that acknowledge the complexity of stimuli-responsive and biomimetic materials.

Looking forward, AI-guided design stands to accelerate every phase of this pipeline. Machine learning and deep learning models can predict formulation outcomes—particle size, drug loading, release kinetics—and recommend optimal polymer–ginsenoside pairings with minimal experimental rounds. Evolutionary and Bayesian optimization algorithms, integrated within microfluidic synthesis platforms, promise rapid, automated refinement of carrier parameters. Molecular simulations augmented by ML ranking will streamline excipient selection, while PBPK–ML frameworks can anticipate *in vivo* biodistribution and optimize targeting ligands. By embedding these AI methodologies into a function-centered roadmap—solubility enhancement, tumor targeting, stimuli responsiveness—researchers can iterate from concept to candidate within weeks rather than months.

Crucially, realizing this vision demands interdisciplinary collaboration: computational scientists to develop and validate predictive models; materials engineers to translate algorithms into manufacturable processes; pharmaceutical toxicologists to ensure safety and regulatory compliance; and TCM experts to align design parameters with traditional usage and clinical needs. Coupling AI’s data-driven insights with domain expertise will not only refine ginsenoside carriers but also establish a template for delivering other challenging natural products. As the field converges on standardized data repositories, open-source AI tools, and harmonized reporting guidelines, the prospect of safe, effective, and personalized ginsenoside therapeutics moves from aspiration to imminent reality—heralding a new era in which ancient remedies are empowered by cutting-edge science.
